# PVA Films with Mixed Silver Nanoparticles and Gold Nanostars for Intrinsic and Photothermal Antibacterial Action

**DOI:** 10.3390/nano11061387

**Published:** 2021-05-25

**Authors:** Pietro Grisoli, Lorenzo De Vita, Chiara Milanese, Angelo Taglietti, Yuri Diaz Fernandez, Margaux Bouzin, Laura D’Alfonso, Laura Sironi, Silvia Rossi, Barbara Vigani, Paola Sperandeo, Alessandra Polissi, Piersandro Pallavicini

**Affiliations:** 1 Department of Drug Sciences, Università di Pavia, v. Taramelli 12, 27100 Pavia, Italy; silvia.rossi@unipv.it (S.R.); barbara.vigani@unipv.it (B.V.); 2 Department of Chemistry, Università di Pavia, v. Taramelli 12, 27100 Pavia, Italy; lorenzo.devita01@universitadipavia.it (L.D.V.); chiara.milanese@unipv.it (C.M.); angelo.taglietti@unipv.it (A.T.); 3 Open Innovation Hub for Antimicrobial Surfaces, Surface Science Research Centre, Department of Chemistry, National Biofilm Innovation Centre, University of Liverpool, Liverpool L69 3BX, UK; Yuri.Diaz-Fernandez@liverpool.ac.uk; 4 Department of Physics “G. Occhialini”, Università Milano-Bicocca, Piazza della Scienza 3, 20133 Milan, Italy; margaux.bouzin@unimib.it (M.B.); laura.dalfonso@unimib.it (L.D.); laura.sironi@unimib.it (L.S.); 5 Department of Pharmacological and Biomolecular Sciences, University of Milano, via Balzaretti 9, 20133 Milan, Italy; paola.sperandeo@unimi.it (P.S.); alessandra.polissi@unimi.it (A.P.)

**Keywords:** photothermal effect, gold nanostars, silver nanoparticles, antibacterial materials, PVA, photothermal film

## Abstract

PVA films with embedded either silver nanoparticles (AgNP), NIR-absorbing photothermal gold nanostars (GNS), or mixed AgNP+GNS were prepared in this research. The optimal conditions to obtain stable AgNP+GNS films with intact, long lasting photothermal GNS were obtained. These require coating of GNS with a thiolated polyethylene glycol (PEG) terminated with a carboxylic acid function, acting as reticulant in the film formation. In the mixed AgNP+GNS films, the total noble metal content is <0.15% *w*/*w* and in the Ag films < 0.025% *w*/*w*. The slow but prolonged Ag^+^ release from film-embedded AgNP (8–11% of total Ag released after 24 h, in the mixed films) results in a very strong microbicidal effect against planktonic *Escherichia coli* and *Staphylococcus aureus* bacterial strains (the release of Au from films is instead negligible). Beside this intrinsic effect, the mixed films also exert an on-demand, fast hyperthermal bactericidal action, switched on by NIR laser irradiation (800 nm, i.e., inside the biotransparent window) of the localized surface plasmon resonance (LSPR) absorption bands of GNS. Temperature increases of 30 °C are obtained using irradiances as low as 0.27 W/cm^2^. Moreover, 80–90% death on both strains was observed in bacteria in contact with the GNS-containing films, after 30 min of irradiation. Finally, the biocompatibility of all films was verified on human fibroblasts, finding negligible viability decrease in all cases.

## 1. Introduction

The use of AgNP as antibacterial agents is well known and documented by more than 14,000 published papers while this article is under preparation (March 2021) [1]. AgNP used as antibacterial agents are typically spheres with diameter of 5–20 nm [2]. While in most cases the antibacterial studies imply their use as colloidal solutions [2], many examples also describe AgNP monolayers as coatings for antibacterial surfaces [3,4,5,6], AgNP dispersed in hydrogels [7] and AgNP embedded in solid films. The latter case comprises the use of such materials for food packaging [8,9], water filtration [10,11] and wound dressing [12,13]. In all these materials, the antibacterial action of AgNP is due mainly to their slow surface oxidation, resulting in the prolonged release of the antimicrobial Ag^+^ ion [2], although an additional nanomechanical effect is sometimes observed, exerted by the disruptive contact of the high-energy AgNP surface with bacterial membranes [4]. While all these are *intrinsic* antibacterial materials, *switchable* ones have recently emerged. In these, gold nanoparticles (AuNP) are typically used. While AuNP do not have significant intrinsic antibacterial properties, they can be exploited to obtain an on-demand local hyperthermal microbicidal action. AuNP have intense, large LSPR absorption bands. Excitation of AuNP (e.g., with a laser source) in the wavelength range of such bands results in thermal relaxation, with a local T increase capable of killing bacteria. This is particularly interesting if considering that low laser intensities can be used (e.g., <0.32 W/cm^2^, the ANSI imposed limit for human skin treatment at 800 nm [14,15]) to obtain a 5–10 °C increase with respect to a given T, including of course that of the human body. Moreover, using properly shaped nanoparticles, LSPR absorptions fall in the near-IR (NIR) [16,17] and irradiation can be carried out in the first biotransparent window (750–900 nm) [18], allowing treatments on an infected zone that avoid undesired hyperthermia of the eventually irradiated nearby healthy tissues. Colloidal solutions of gold nanorods [19], hydrogel-embedded gold nanorods [20], glass surfaces coated with monolayers of gold nanostars (GNS) [21] and PVA-embedded GNS [22] have recently been demonstrated to be efficient in thermally killing Gram-negative (*E. coli* [20,22], *Acinetobacter baumannii* [20], *Pseudomonas aeruginosa* [20] and *Klebsiella pneumoniae* [20]) and Gram-positive (*S. aureus* [19,20,21], *Propionibacterium acnes* [19] and *Staphylococcus epidermidis* [20]) bacterial strains. Materials capable of exerting both an intrinsic and a photothermal on-demand antibacterial action are a valuable evolution of the described state of the art. Besides the prolonged protection against bacterial colonization exerted by AgNP, the photothermal bactericidal action of AuNP can be turned on in case of more severe conditions, e.g., if a biofilm is formed on the surface of an indwelling device or in a chronic wound.

**Scheme 1:**
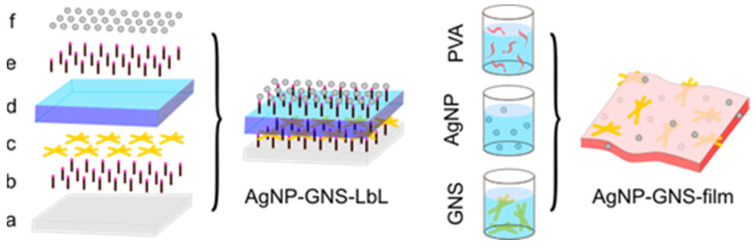
**Left**: the multistep approach required to obtain AgNP-GNS-LbL, i.e., materials with a monolayer of AgNP and an underlying layer of photothermal GNS, as described in [31]. **Right**: the single-step approach to obtain the films described in this paper.

When working with AuNP featuring LSPR bands in the biotransparent window, this action can be carried out through-tissues (in the case of indwelling devices and prosthesis) and avoiding to damage the surrounding skin and deeper tissues (in the case of wounds). However, it may be a tricky issue to obtain the coexistence of AgNP and AuNP (especially of non-spherical shape) in a colloidal solution, in a solid material or in a mixed monolayer on a surface. The different intrinsic stability [23,24] and the different required synthetic routes [6,16,21,25,26] for the two NP types are adverse factors. Moreover, the strikingly different shape, surface energy, oxidation potential and solubility of spherical AgNP of 5–20 nm diameter with respect to larger, non-spherical AuNP (e.g., GNS or nanorods) can lead to interparticle Ostwald ripening processes [27] and underpotential Ag^+^ deposition on gold [28], leading to Ag-promoted Au nanoparticles reshaping [29,30]. We recently prepared glass slides bearing both a GNS monolayer and a separate AgNP monolayer on top of it [31], sketched as AgNP-GNS-LbL in Scheme 1. However, a tricky, time-consuming layer-by-layer approach was necessary, from pre-prepared AgNP and GNS colloidal solutions. Five sequential steps were in fact required (Scheme 1, left), as a SiO_2_ barrier (d) was used to keep AgNP (f) and GNS (c) physically separated. Moreover, functional silane monolayers (b and e) were also needed to graft GNS to the underlying glass surface (a) and AgNP to the SiO_2_ layer (d). This material exerted a strong intrinsic antibacterial effect due to the spherical AgNP (d = 8 nm) on the top layer, plus a strong hyperthermal antibacterial effect when the LSPR band of the embedded GNS was excited with an 800 nm laser source.

In the frame of our recent interest for nanoinorganic-based antibacterial products to be used in the treatment of wounds [32,33], we have now prepared, for the first time, a flexible film containing both small spherical AgNP and photothermally active GNS, i.e., a film capable of both intrinsic and switchable antibacterial action. We adopted a straightforward approach, as films containing both AgNP and GNS (AgNP-GNS-film in Scheme 1) are prepared in a single casting step, after mixing aqueous solutions of a polymer with adequately coated GNS and AgNP. PVA (polyvinyl alcohol) was chosen as the polymer, due to its low toxicity, biocompatibility and easy film-forming procedures [34,35]. PVA films are also strongly hydrophilic, a potentially useful property in removing exudate during wound-healing processes [36]. In this paper, we describe the preparation, the AgNP and GNS coatings with which film stability is obtained, the Ag^+^ release properties, the photothermal response, the morphology and the tensile properties of such PVA-based films. Their intrinsic and photothermal antibacterial action was measured both on planktonic and on film-inoculated *E. coli* and *S. aureus* bacterial strains, and the biocompatibility of such films was verified on human fibroblasts.

## 2. Materials and Methods

Silver nitrate ACS reagent, ≥99.0%; pectin from citrus peel (galacturonic acid ≥ 74.0%, dried basis); Triton™ X-100 (laboratory grade); gold(III) chloride solution 99.99% (30 wt. % in dilute HCl); sodium borohydride ≥ 98.0%; L-ascorbic acid ≥ 99.0%; poly(ethylene glycol) methyl ether thiol (PEG-SH, mw 2000); *O*-(3-Carboxypropyl)-*O*′-[2-(3-mercaptopropionylamino)ethyl]-polyethylene glycol (HS-PEG-COOH, mw 3000); poly(vinyl alcohol) Mw 89,000–98,000, 99+% hydrolysed; poly(ethylene glycol) (mw 200); citric acid ACS reagent ≥ 99.5%; sodium hydroxide ≥ 98%, pellets; nitric acid ≥ 65%; and hydrochloric acid ≥ 37% have all been bought by Merck Life Sciences, Milano, Italy, and used without further purification.

All the glassware used in the syntheses was pre-treated with aqua regia and then by washing three times with bidistilled water in an ultrasound bath for 5 min.

Centrifugation was carried out using the ultracentrifuge Hermle Z366 with polypropylene 10 mL tubes.

Measurement of pH was carried out with a XS Instruments pH-meter (pH 50 model) with a Thermo Scientific Orion 91022 BNWP combined glass electrode. Electrode calibration was carried out before measurements, with solutions buffered at pH = 4 and 7 or at pH = 7 and 10.

Absorption spectra were recorded on a HP8453 spectrophotometer, either in solution in 1 mm and 1 cm glass cuvettes or directly on films, by means of a dedicated sample holder.

### 2.1. TEM Imaging

Solutions of AgNP and GNS were diluted 10–100 times with bidistilled water and 10 μL were deposited on nickel grids (300 mesh) covered with a Parlodion membrane and dried in a desiccator. Images were taken using a JEM-1200 EX II 140 instrument (JEOL, Basiglio (MI), Italy).

### 2.2. SEM Imaging

Morphology characterization was carried out by scanning electron microscopy (SEM). Samples were sputtered with gold and submitted to scanning electron microscope (SEM) Zeiss EVO MA10 (Carl Zeiss, Oberkochen, Germany). The images were acquired at high voltage (20 kV), in high vacuum, at room temperature and at different magnifications.

### 2.3. Thermograms

We employed a FLIR E40 thermal camera with FLIR Tools+ dedicated software for data acquisition and analysis. Thermal images were 320 × 240 pixels, and for each thermogram, a ROI was defined, which comprised the laser-irradiated area. From data analysis, we determined the maximum temperature inside the ROI (±0.1 °C accuracy) for each thermal image. In a typical thermogram, a thermal image was acquired every 0.25 or 0.5 s for 60–240 s. The used laser source (808 nm) had a beam waist of 1.0 cm and power tunable between 0 and 200 mW.

### 2.4. Ag and Au Release

Briefly, 90 mg portions of each film were suspended in 3.0 mL bidistilled water in a stoppered vial and incubated at 37 °C for 1, 5 and 24 h, after which time, 1.0 mL was picked up and treated with 1.0 mL aqua regia and allowed to react for 1 h. Then, 3.0 mL bidistilled water was further added, and the sample was analysed by ICP-OES on a Optima 3300 DV instrument (Perkin Elmer, Milano, Italy). The “refresh + 24 h” data were obtained by picking up the films from vials after 24 h and dipping them in 3.0 mL of bidistilled water for further 24 h. Then, 1.0 mL of the solution was picked up and treated as described.

### 2.5. Mechanical Properties

The mechanical properties of films were assessed by means of a TA.XT plus Texture Analyzer (Stable Micro Systems, Godalming, UK), equipped with a 5 kg load cell. Before testing, film thickness was measured by means of a Sicutool 3955 G-50 (Sicutool, Milan, Italy) apparatus. Each film was cut (1 × 3 cm) and, then, clamped on A/TG tensile grips probe; an initial distance of 1 cm between the grips was set. The upper grip was raised at a constant speed of 5 mm/s up to a distance of 30 mm, corresponding to 300% elongation. Maximum deformation force (F_max_), forces at different deformations (F50, F100, F150 and F200 at 50%, 100%, 150% and 200% elongation, respectively) and deformation work (W_max_) were measured.

### 2.6. Syntheses

#### 2.6.1. AgNP

Pectin-coated AgNP were prepared according to a described procedure [32,33]. Briefly, in a typical preparation, 0.3 g pectin from citrus peel was dissolved in 30 mL bidistilled water by stirring 30 min at 60 °C. When dissolution was complete, 1.0 mL of 0.03 M AgNO_3_ in water was added, quickly followed by 3.0 mL of a standard 0.5 M NaOH water solution. The solution was maintained at 60 °C for 14 h. Yellow-orange AgNP were obtained, with a pH of 10.5–11.4. For preparations needing a neutral pH, this was regulated at 7.0–7.5 by microadditions of 0.1 M HNO_3_.

#### 2.6.2. GNS

The syntheses of GNS were carried out according to our described method [37]. Briefly, in a typical synthesis, first a seed solution was obtained preparing 5.0 mL of 0.2 M Triton X-100 to which 5.0 mL HAuCl_4_ 0.445 mM was added, rapidly followed by 600 μL of ice-cooled 0.01 M NaBH_4_ in water. The obtained seed solution (pink-orange colour) was maintained in an ice bath and used within 1 h. For the growth solution, 50 mL of 0.2 M Triton X-100 was put in a 250 mL flask under magnetic stirring. In a fast sequence, we added 2.5 mL AgNO_3_ 0.004 M, 50 mL HAuCl_4_ 0.89 M, 1.60 mL ascorbic acid 0.0788 M and, finally, 36 μL of a freshly prepared seed solution. Stirring was stopped, and after 2 h, the GNS solution was considered fully reacted (blue-black colour). Coating with HS-PEG (mw 2000) or HS-PEG-COOH (mw 3000) was carried out by dissolving 4.0 mg or 6.0 mg, respectively, in 2.0 mL bidistilled water; this solution was added to the freshly prepared GNS solution at room temperature and allowed to react for 5 h. After this time, pegylated GNS were purified by ultracentrifugation, subdividing the whole volume in 10 mL portions treated at 13,000 rpm (15,870 rcf) for 25 min. This led to the separation of GNS pellets on the bottom of the centrifuge tubes. The colourless supernatant was removed, the pellet was redissolved in 10 mL bidistilled water (ultrasonication was applied for 30 s) and the whole process was repeated two more times to obtain a solution of pure pegylated GNS. Concentrated GNS solutions were obtained by redissolving the pellet, in the last cycle, in a smaller bidistilled water volume.

#### 2.6.3. PVA Films

In a 50 mL beaker, 10 mL of 6% *w*/*w* PVA solution in water were prepared, by heating at 90 °C until complete solubilization (the beaker was covered with a watch glass). The solution was then allowed to return to RT; then, 66 mg of PEG200 was added and the solution was stirred for 1 h. In the case of films reticulated with citric acid, we carried on the following additions: (i) nothing (**film-blank**), (ii) 1.0 mL of 1% *w*/*w* pectin solution (**film-pec**), (iii) 1 mL of AgNP solution at pH ~ 7 (**film-Ag**), (iv) 0.5 mL concentrated GNS@PEG solution (**film-GNS**), (v) 1.0 mL 1 mL of AgNP solution at pH ~ 7 and 0.5 mL concentrated GNS@PEG solution (filmMIX-Ag/GNS); then, for cases (i)–(v) after stirring at room temperature for 3 h, 66 mg citric acid was added and allowed to solubilize. In the case of reticulation with GNS coated with HS-PEG-COOH (PEG-C), **film-GNS-C** was prepared by adding 0.5 mL of concentrated GNS@PEG-C to the PVA/PEG200 solution, and filmMIX-Ag/GNS-C by adding 1 mL of AgNP at pH ~ 7 and 0.5 mL concentrated GNS@PEG-C to the PVA/PEG200 solution; after this, stirring was continued at RT for 3 h with no further additions.

Finally, all films were obtained by pouring their solution in a Petri dish (diameter = 9 cm), heating in an oven at 130 °C for 5 min and then allowing to stay and dry at RT in air for 4 days, after which time they can be easily peeled off the Petri dish.

### 2.7. Microbiological Experiments

#### 2.7.1. Antimicrobial Activity on Planktonic cells

We used *Staphylococcus aureus* ATCC 6538 and *Escherichia coli* ATCC 10356 (Thermo Fisher Diagnostic S.P.A, Rodano(MI), Italy). Tryptone soya Agar (TSA) and tryptone soya broth (TSB) were purchased from Oxoid (Basingstoke, UK).

For the determination of the ME on planktonic cells, bacteria were grown overnight in TSB at 37 °C. The bacteria cultures were centrifuged at 2000 rpm for 20 min to separate cells from broth and then suspended in phosphate buffer saline (PBS, pH 7.3). The suspension was diluted to adjust the number of cells to 1 × 10^7^–1 × 10^8^ CFU/mL. A portion of each film (30 mg/mL) was added to the suspensions of microorganisms. For each microorganism used, a suspension was prepared in PBS without PVA films and used as control. Bacterial suspensions were incubated at 37 °C. Viable microbial counts were evaluated after contact for 5 and 24 h with PVA films and in control suspensions; bacterial colonies were enumerated in Tryptone Soya Agar after incubation at 37 °C for 24 h. The microbicidal effect (ME value) was calculated for each test organism, and contact times were calculated according to the following equation [38]:

ME = logN_C_ − logN_A_, (1)

where N_C_ is the number of CFU of the control microbial suspension and N_A_ is the number of CFU of the microbial suspension in presence of the PVA film.

For the determination of the film contact effect, microbial suspensions were filtered with an initial inoculum of approximately 1–2 × 10^7^ CFU/mL on filter membranes of cellulose acetate with a porosity of 0.22 μm. The membranes were then deposited on Petri plates containing TSA, suitable for the growth of microorganisms selected. The filter membranes were covered with PVA films for 5 and 24 h contact times. Similarly, plates were prepared containing membrane filters without PVA films (control). After contact, the filter membranes were recovered and washed by suspending them in sterile water with a standardized method, and at the end of the washing procedure, dilutions of the microbial suspensions were made to determine the microbial content with subsequent plating in TSA in order to count the viable cells and calculate the contact, apparent microbicidal effect (ME) as:

ME = logN_C_ − logN_d_, (2)

where N_C_ is the number of colonies forming unit (CFU) of the control and N_d_ is the number of CFU in the cases of contact with the films.

#### 2.7.2. Photothermal Antimicrobial Activity

*E. coli* MG1655 and *S. aureus* ATCC 6538P strains were routinely grown in LB (Bacto Tryptone 10 g/L; yeast extract 5 g/L; NaCl 10 g/L; Difco; Franklin Lakes, NJ, USA) agar plates, incubating at 37 °C. For antibacterial effect assessment, a single bacterial colony was inoculated in 5 mL of liquid LB broth and incubated at 37 °C under aeration for 16–18 h. Following incubation, the bacterial culture was diluted 1:10 in the same medium to adjust the bacterial concentration to approximately 1–5 × 10^8^ CFU/mL. We then followed the method that we have already described [39]. Briefly, films were cut to fit laser beam size (~1 cm diameter) and placed in Petri dishes with a cover glass bottom (MatTek, Ashland, MA, USA). An amount of 20 μL of diluted bacterial suspension was inoculated on the top of the glasses, and the films were gently air-dried under a laminar flow hood. The films were irradiated with 800 nm laser light (0.30 W/cm^2^ on the sample plane) and different durations of irradiation (0, 15 and 30 min). Irradiated and non-irradiated (control) samples were stained with Film Tracer Live/Dead viability kit (L10316, Invitrogen, Carlsbad, CA, USA) based on the use of the SYTO^®^ 9 and propidium iodide stains mixture in an appropriate concentration ratio (0.167 or 3.34:20) to ensure that the bacteria with intact membranes (live) stain fluorescent green (SYTO^®^ 9), whereas only the bacteria with damaged membranes (dead) are stained red with propidium iodide. The stained samples were analysed with a Leica SP5 TCS confocal microscope using a 20× dry objective (HC PL FLUOTAR 20 × 0.5, dry, Leica, Wetzlar, Germany). At least four z-stacks of raster scanned images were collected from three distant regions in the film by using the 488 nm argon ion laser emission in both spectral intervals (510–580 and 590–700 nm, where the bleed through of the green dye at the concentration used for the staining experiments is negligible). Each image was acquired with either 512 × 512 or 1024 × 1024 pixels and 400 Hz of line scan frequency, with fields of view ranging from 64 μm × 64 μm to 110 μm × 110 μm. Z-stacks were acquired by taking planes every 1.0 μm. The images were analysed by employing a threshold filter (Fiji, version 2.0.0-rc-43/1.52n, NIH) and measuring the percent area in the red (A_dead_) and green (A_live_) channels.

### 2.8. Cytotoxicity Tests

An indirect test was performed to evaluate film cytotoxic effect. Dried films (1 cm^2^) were soaked in 2 mL of complete culture medium (CM), at 37 °C, for 24 h under mild stirring (60 rpm). After 24 h, the “conditioned” medium was recovered and put in contact with a fibroblast monolayer. Briefly, cells were seeded on 96-well plates (3.5 × 10^4^ cells in 200 μL of CM/well) and incubated (37 °C and 5% CO_2_) for 24 h in order to reach semi-confluence. 200 μL of each sample (conditioned medium) was put in contact for 24 h with cells; CM was used as reference. After incubation, an MTT assay was performed. Samples and reference were removed from the 96-well plate, and cell monolayers were washed with PBS; subsequently, 50 μL of MTT 7.5 μM in 100 μL of DMEM without phenol red was added to each well and incubated for 3 h (37 °C and 5% CO_2_). Finally, 100 μL of DMSO, used as solubilisation agent, was added to each well. In order to promote the complete dissolution of formazan crystals, obtained from MTT dye reduction by mitochondrial dehydrogenases of alive cells, absorbance of the solution was measured by means of an iMark^®^ Microplate reader (Bio-Rad Laboratories S.r.l., Hercules, CA, USA) at a wavelength of 570 and 690 nm (reference wavelength) after 60 s of mild shaking. Results were expressed as % cell viability by normalizing the absorbance measured after contact with each sample with that measured for CM. Twelve replicates were performed for each sample.

## 3. Results and Discussion

### 3.1. AgNP and GNS

**Figure 1:**
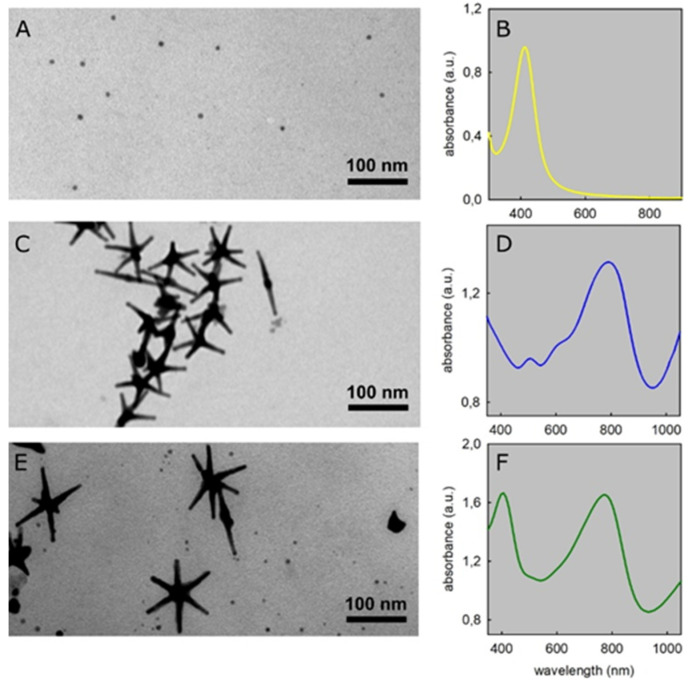
(**A**,**C**,**E**) TEM images obtained from solutions of AgNP, GNS and 1:2 AgNP:GNS solution mixtures, respectively. (**B**,**D**,**F**) Corresponding absorption spectra. Please note that spectrum (**B**) is displayed on the 350–900 nm range, while spectra (**D**,**F**) on the 350–1050 nm range.

Colloidal solutions of AgNP were prepared from Ag^+^ (nitrate salt) by treatment with pectin in aqueous basic solution. The reduction to Ag(0) proceeds thanks to the oxidation of the diol moiety of the galacturonic acid units of pectin, with consumption of hydroxide anions [32,33]. This synthesis was recently introduced by us, as it allows the fast, reproducible preparation of spherical AgNP with 8.0(±2) nm diameter (Figure 1A), with a ~100% Ag^+^ to Ag(0) conversion yield (residual Ag^+^ < 0.8% of total Ag) [32,33]. In this work, we adopted an established set of optimal conditions, using 1% *w*/*w* pectin in water, 1.0 mM AgNO_3_ and a starting pH in the 10.5–11 range, at T = 60 °C. Under these conditions, the galacturonic acid units of the pectin chains are in large excess with respect to Ag^+^, thus maintaining AgNP stable with respect to oxidation by O_2_ [23,24,32]. Moreover, pectin also efficiently stabilizes AgNP against aggregation [32]. The obtained AgNP have a sharp LSPR absorption band at λ_max_ 412 nm, Figure 1B, typical of small, spherical AgNP [40,41,42], imparting to their solutions the characteristic yellow-brown colour.

GNS were also synthesized, according to a method developed in our laboratory [37] and later exploited by other authors [43,44]. This is a seed-growth process in water, with the Triton X-100 surfactant as the protecting and shape-directing agent, ascorbic acid as the reductant, HAuCl_4_ as the starting Au compound and small quantities of AgNO_3_ required as a further shape-directing agent. After growth, GNS are stabilised by grafting a thiolated polyethylene glycol (HS-PEG) of high molecular weight on their surface. In this paper, we used both HS-PEG (mw 2000, methoxy-terminated) and HS-PEG-COOH (mw 3000), the latter bearing a thiol on one end and a carboxylic acid function on the other end of the chain. PEG grafting on GNS surface is compulsory. Triton X-100 is cytotoxic and must be removed by repeated ultracentrifugation cycles, which would result in a severe product loss if uncoated GNS were used. Moreover (vide infra), mixing as-prepared GNS with AgNP induces a fast reshaping of the previous, with dissolution of the latter. On the contrary, GNS coated with a HS-PEG have optimal stability and solubility, surfactant is completely removed by repeated ultracentrifugation steps [45] and they are stable when mixed with AgNP. These GNS have typically 2–6 sharp branches protruding from a core. Under the synthetic conditions used in this paper, GNS with 5–6 branches are prevalently obtained, with a tip-to-tip maximum distance of 80–100 nm, see Figure 1C (see Supplementary Materials, Figure S1a,b, for larger TEM images). These GNS have multiple LSPR absorption bands, dominated by two large intense peaks (peak 1 and peak 2) whose λ_max_ can be tuned during synthesis between 750 and 1100 nm and between 1200 and 1600 nm, respectively [37]. Figure 1D reports the absorption spectrum in the 350–1050 nm range of a typical GNS colloidal solutions used in this paper, with peak 1 λ_max_ at 790 nm (peak 2 is out of range). The low intensity peak at 520 nm is due both to spherical by-products and to the transversal oscillation of the conduction electrons in the branches, typical of all elongated gold nanoparticles [26,46]. A weak shoulder at 650 nm can also be noticed in Figure 1D, which is attributed to a minor population of partially grown nano-objects.

Although complex spectra and a multiple-shape population may be unsatisfactory on an aesthetic point of view, the synthesized GNS colloidal solutions have all the needed features for the aim of this work: a strong, large LSPR absorption falling in the first biotransparent window, with λ_max_ near to the typical laboratory NIR laser source wavelength, i.e., 800 nm.

### 3.2. AgNP + GNS Mixtures and Films Preparation

Solutions containing pectin-coated AgNP and pegylated GNS were prepared by mixing the pre-prepared individual colloidal solutions. The as-synthesized colloidal solutions of AgNP have a total Ag concentration of 8.82 × 10^−4^ M, corresponding to all the starting Ag^+^ that is converted into Ag(0) (yield is >99%). The pH value is 9.5–10. For GNS, the starting total Au concentration is 4.27 × 10^−4^ M [37,45], but after PEGylation and repeated purification by ultracentrifugation, we typically obtain solutions with total Au concentration in the 2.5–3.0 × 10^−4^ M range and pH 5.5–6. Adding a given volume of such GNS solutions to comparable volumes of AgNP gives mixed solutions in which the LSPR band of GNS has a too low absorption with respect to that of AgNP. We thus modified the workup after PEGylation, by concentrating the GNS solutions to total Au = 8.68 ± 0.52 × 10^−3^ M (1.71 ± 0.10 mg/mL, data determined on the average of 3 preparations by ICP-OES analysis, see Materials and Methods section for details). Data refer to GNS coated with HS-PEG (GNS@PEG). Almost identical results were found for GNS@PEG-C, i.e., using HS-PEG-COOH as coating (total Au = 8.53 ± 0.91 × 10^−3^ M, 1.68 ± 0.18 mg/mL). It has also to be pointed out that the Ag^+^ ions added to promote the GNS asymmetric growth are incorporated in the GNS body during synthesis, with a total Ag atom concentration typically of 18–20% vs. Au [37,45]. Accordingly, the Ag content has also been analysed in the GNS concentrated solutions, finding 1.94(±0.16) × 10^−3^ M (0.210 ± 0.018 mg/mL) for GNS@PEG and 1.86(±0.25) × 10^−3^ M (0.199 ± 0.028 mg/mL) for GNS@PEG-C. When preparing mixed solutions, a linear dependence of the GNS absorbance at their λ_max_ is observed by adding increasing quantities of concentrated GNS solution to AgNP (Supplementary Materials, Figure S2). We adopted the volume ratio AgNP:GNS 2:1 as a standard, as it gives an almost identical absorbance for the two LSPR peaks. A representative spectrum of the obtained deep green mixed solutions is shown in Figure 1F (Supplementary Materials, Figure S3a, for a photograph of the solution). A TEM image obtained from this solution is shown in Figure 1E (Supplementary Materials, Figure S1c for a larger image). However, such mixed solution is not stable for the long period. A decrease of the AgNP LPSR band is noticed in 1–7 days range, with the simultaneous blue shift of the GNS LSPR (Supplementary Materials, Figure S3b,c). Although it has not been further investigated, this can be explained with the slow reduction and deposition of the equilibrium concentration of Ag^+^ from AgNP (10 ppm [32]) on the highest energy portions of the GNS surface (corresponding to the sharp edges and tips of branches). This leads to AgNP consumption and GNS reshaping. Ag^+^ reduction is likely due to excess pectin, in a reaction that needs a basic pH [32]. Accordingly, we changed the pH of the AgNP solution to 7.0–7.2 by microadditions of 0.1 M HNO_3_ prior to mixing with GNS. Stable mixed solutions were obtained (Supplementary Materials, Figure S3d). It has to be stressed that pectin coating for AgNP, PEG coating for GNS and pH regulation are of utmost importance. As a comparison, we spectroscopically checked the stability of mixed solutions containing standard citrate-coated AgNP (d = 7–8 nm, prepared as in refs [6] and [25]) and as-prepared GNS from Triton X-100. The complete disappearance of the AgNP plasmon band (394 nm) was observed in 6 h, with a blue shift of GNS LSPR band (Δλ = −7 nm), see Supplementary Materials, Figure S4.

Films were prepared with slight modifications of an established procedure [47] by casting aqueous solutions containing PVA (mw 89,000–98,000, 6% *w*/*w*), a short chain non-functional PEG as a plasticizer (mw 200, 66 mg, 11% of the PVA mass) and exploiting the ester formation reaction [48] between the poly-carboxylate units of a cross-linker and the –OH functions of PVA. Small volumes (0.5–1.0 mL) of pectin in water (1% *w*/*w*), of aqueous solutions of AgNP (pH 7), of GNS@PEG and of GNS@PEG-C were added to 10 mL of the PVA solution, obtaining the set of film-forming solutions listed in Table 1.

Citric acid (CA) was added as the cross-linker in all cases (66 mg, 11% of the PVA mass), except with GNS@PEG-C. In the latter case, the terminal –COOH functions of the grafted HS-PEG-COOH coater acted as cross-linkers. Casting was obtained by pouring a given volume of the solutions (10.0–11.5 mL, depending on the film type) in round flat Petri dishes (d = 9 cm), heating at 130 °C for 5 min and then keeping the films at room temperature for 4 days, thus allowing complete evaporation of excess water. After this time, film disks were easily peeled off from the Petri dishes and kept in the dark for all further characterization. Knowing the Ag and Au concentrations in the AgNP and GNS solutions, the total Ag and Au content in each film can be calculated, as listed in Table 1. In the mixed films **film/mix/Ag/GNS** and **film/mix/Ag/GNS-C**, the total Au content is identical to that of the corresponding GNS-only films, while the Ag content is higher than in all other cases, due to the contribution of both AgNP and of the Ag content of GNS. The approximate total mass of a standard film disk (water excluded) can also be calculated, as 600 mg PVA, 66 mg PEG200 and 66 mg citric acid are added in all cases (total mass 732 mg), except in films containing GNS@PEG-C, where citric acid is not added (total mass 666 mg). A further 10 mg contribution comes from pectin in the films with added AgNP. As it can be seen from Table 1, the total noble metal content is in all cases <1 mg, i.e. negligible with respect to overall mass. In particular, the content of the potentially toxic Ag element is extremely low (<0.025% *w*/*w*). Weighting the film disks after the 4 days standard drying time (Table 1) shows that the water content is in all cases <2% of the total film mass.

### 3.3. Effect on AgNP and GNS of PVA Addition and Film Formation

**Figure 2:**
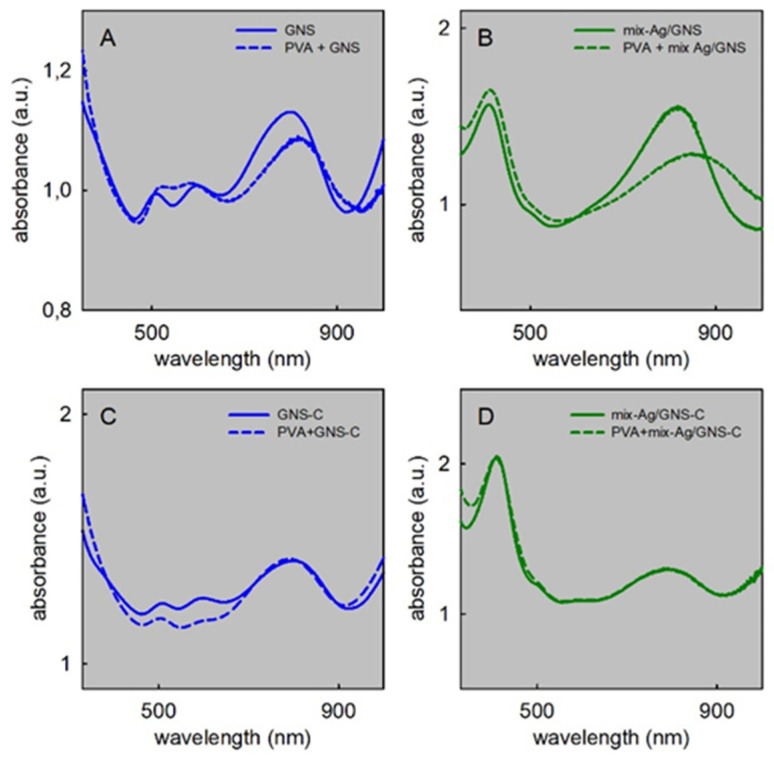
Absorption spectra of nanoparticles colloidal solutions before (solid lines) and after (dashed lines) addition of PVA: (**A**) GNS, (**B**) 1:2 AgNP:GNS mixture, (**C**) GNS-C and (**D**) 1:2 AgNP:GNS-C mixture.

Spectral changes take place on mixing GNS@PEG solutions with PVA. A red shift and an intensity decrease of the GNS LSPR band was observed immediately after mixing, both in the presence or absence of AgNP (at pH 7), Figure 2A,B. On the contrary, in the mixed solution, the LSPR band of AgNP (λ_max_ 412 nm) does not change significantly its position or shape.

The absorption spectra of AgNP in the presence of PVA, PVA + PEG200 and PVA + PEG200 + citric acid have also been recorded for comparison, showing similarly negligible variations of the LSPR band (Supplementary Materials Figure S5; the absorption spectra of citric acid and PEG200 are also displayed in the same figure for comparison, and they do not have absorptions in the 250–1100 nm range). Very interestingly, the absorption spectrum of GNS@PEG-C does not change when PVA is added, Figure 2C. The same is observed for GNS@PEG-C in the presence of AgNP in the mixed solutions, Figure 2D. We hypothesize that with GNS@PEG, the added PVA is allowed to interact with the gold surface and displaces the surface-confined residual water molecules, as the easy access of molecular species to the surface of HS-PEG-coated GNS has already been observed [49]. The higher PVA refractive index (1.4748) compared with that of water (1.33) and the high sensibility of the NIR LSPR bands of GNS to refractive index changes [16] fit well in this picture. In the case of GNS@PEG-C, the absence of an LSPR band shift is attributed to the presence of the carboxylate functions at the remote end of the HS-PEG-COOH coating. The esterification reaction among the –COOH groups and the PVA hydroxyl groups causes the reticulation of the PVA films when no citrate is added. While the full completion of reticulation could be slow, we can hypothesize that is starts immediately, condensing the –OH groups of PVA to the remote COOH functions of GNS@PEG-C and thus preventing the contact of PVA with the gold surface. According to the published data of % weight of HS-PEGs on these GNS (~10%) [21], a 500,000-fold excess of –CH_2_CH(OH)– groups (from PVA) vs. –COOH groups (from HS-PEG-COOH) can be calculated in our synthetic conditions. This strongly supports the hypothesis of a fast esterification reaction.

**Figure 3:**
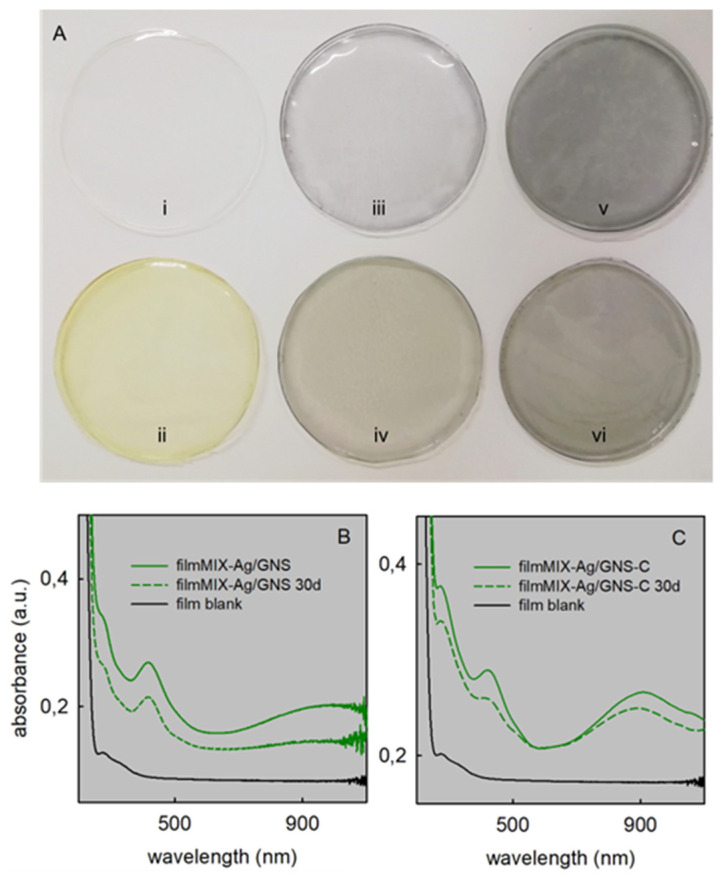
(**A**) Photographs of films (diameter of each disc ~9 cm): (i) **film-blank**, (ii) **film-Ag**, (iii) **film-GNS**, (iv) **filmMIX-Ag/GNS**, (v) **film-GNS-C**, and (vi) **filmMIX-Ag/GNS-C**. (**B**) Absorption spectrum of freshly prepared (green solid line) and 30-days (green dashed line) aged **filmMIX-Ag/GNS** and (**C**) for **filmMIX-Ag/GNS-C**. The absorption spectrum of **film-blank** (black solid line) is added in (**B**,**C**) for comparison.

The stability vs. time of the included AgNP and GNS in the prepared films has also been investigated by absorption spectroscopy. Films are transparent (thickness is in the 68–99 μm range, vide infra) and allow excellent light transmission, while the included nanoparticles are in such concentrations that their absorption bands can be easily observed. The reference, void PVA films (**film-blank**) are colourless, the AgNP-containing films (**film-Ag**) are yellow and the GNS containing films (**film-GNS** and **film-GNS-C**) have a blueish-grey colour (see Figure 3A). As expected, **filmMIX-Ag/GNS** and **filmMIX-Ag/GNS-C** have a dark green/grey colour (Figure 3A). However, both **film-GNS** and **filmMIX-Ag/GNS** display a weaker colour with respect to the films prepared with GSN@PEG-C (**film-GNS-C** and **filmMIX-Ag/GNS-C**) despite the GNS concentration is almost identical (Table 1). Such visual appearance is confirmed by absorption spectra, as the GNS LSPR band in freshly prepared films is weaker for films with GNS@PEG (spectra carried out after the standard 4 days drying period at RT) (see Figure 3B,C in the case of **filmMIX-Ag/GNS** and **filmMIX-Ag/GNS-C**). Moreover, after 30 days at RT and in the dark, **filmMIX-Ag/GNS** displays a very low, flat GNS LSPR band (Figure 3B, dashed spectrum), while **filmMIX-Ag/GNS-C** absorption band changes only slightly after 30 days ageing (Figure 3C, dashed spectrum). The same happens when comparing **film-GNS** and **film-GNS-C** after 30 days ageing (Supplementary Materials, Figure S6). The LSPR band of AgNP in **film-Ag** remains instead well defined after 30 days with no significant changes. The behaviour of films containing GNS could be attributed to a residual mobility of the latter in the formed PVA matrix and to the tendency of PEG-coated Au nanoparticles to segregate in hybrid materials, a process that we have already observed for spherical Au nanoparticles [50]. Segregation and short GNS-GNS distances lead to plasmon hybridization and band flattening [51,52,53]. On the other hand, the films containing GNS@PEG-C profit from a strongly reduced GNS mobility as their coating is itself the reticulating agent. Further investigation of this aspect was out of the goals of this work. To obtain a switchable antibacterial photothermal effect, we need films with stable, intense LSPR bands in the NIR; **film-GNS-C** and **filmMIX-Ag/GNS-C** are perfectly satisfactory on this point of view.

### 3.4. Films Characterization

**Figure 4:**
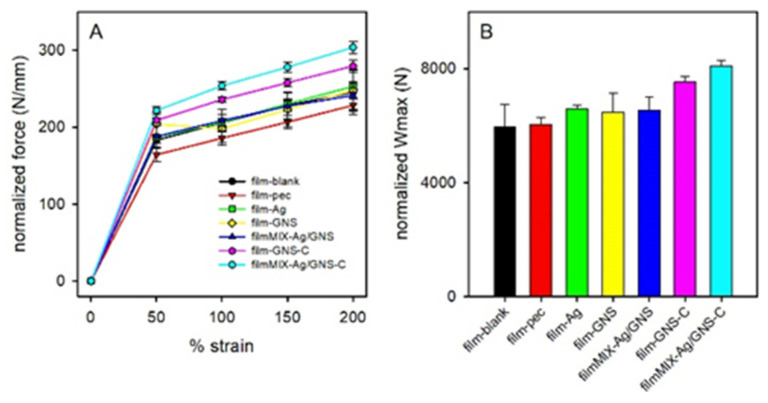
(**A**) Normalized force vs. strain profiles and (**B**) normalized work of deformation. Profiles and bars of the same colour in (**A**,**B**) refer to the same film.

The thickness of the reference **film-blank** (i.e., citrate as cross-linker) is 100 ± 3 μm. All other films with different added components and citrate as cross-linker were obtained with a similar thickness (87–99 μm range). The two films containing GNS@PEG-C are less thick, i.e., 68 ± 4 μm for **film-GNS-C** and 65 ± 3 μm for **filmMIX-Ag/GNS-C**. This indicates a more compact and dense structure when GNS@PEG-C acts as the reticulant. The mechanical properties of the films were also investigated, confirming this hypothesis. The force vs. % strain profiles, reported in Figure 4A, show that all the samples considered are characterized by an increase in the normalized force on increasing % strain.

Such behaviour points out that up to 200% strain, the stress applied does not produce any breakdown in the structures of samples, i.e., films deform without losing their integrity. As for the maximum normalized force, the presence of AgNP does not affect the film mechanical resistance (see comparison among **film-blank**, **film-PEC,** and **film-Ag**). Similarly, the addition of GNS@PEG, alone or combined with AgNP, does not produce any significant change in the film mechanical properties. On the contrary, the incorporation of GNS@PEG-C is responsible for an increase in film mechanical resistance. The normalized tensile work values, calculated as the area under the curve (AUC) force vs. deformation distance (Figure 4B), confirm this behaviour, i.e., an increase in film mechanical resistance is observed for films containing GNS@PEG-C with respect to those cross-linked with citric acid. Noticeably, the film containing a mixture of GNS@PEG-C and AgNP (**filmMIX-Ag/GNS-C**) is characterized by the highest normalized tensile work value, when compared on statistical basis with all the other films considered in the study. Hydrolysed pectin is present as AgNP coater and has both –COO^−^ and –OH groups on its polygalacturonic acid chains. Its role as co-reticulating agent may be hypothesized to explain the observed further resistance increase in **filmMIX-Ag/GNS-C**.

**Figure 5:**
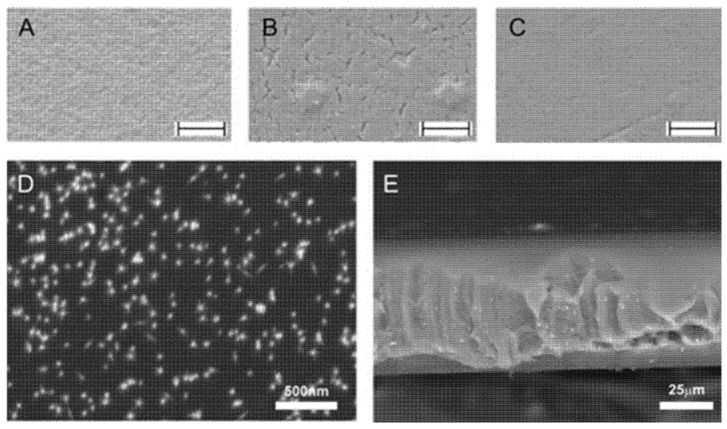
Low-magnification SEM images (20,000×) of **film-blank** (**A**), **filmMIX-Ag/GNS** (**B**) and **filmMIX-Ag/GNS-C** (**C**), scale bar = 1 µm. (**D**) backscattered electron SEM image of the **filmMIX-Ag/GNS-C** surface and (**E**) of its side section; scale bar dimensions are indicated in the figures.

Low-magnification (20,000×) scanning electron microscopy (SEM) imaging was carried out on **filmMIX-Ag/GNS**, **filmMIX-Ag/GNS-C** and **film-blank**. Images in Figure 5A–C show a smoother and more compact surface for **filmMIX-Ag/GNS-C** with respect to **filmMIX-Ag/GNS** (where cracks are large and numerous) and with respect to **film-blank**. This is coherent with the previous observations. Higher magnification SEM images were taken on **filmMIX-Ag/GNS-C** (Supplementary Materials, Figure S7), allowing to observe correctly shaped GNS near the surface (AgNP are too small to be detected on this scale). Backscattered electron imaging of the surface (Figure 5D) and of a section of the film (Figure 5E) shows a homogeneous GNS distribution.

All films are hydrophilic, and when fully dipped in water quickly absorb it up to 200–300% of their mass (1 h), independently on the type of film. Prolonged immersion (>6 h) does not lead to further hydration but partial films disruption is observed. On the mechanically more resistant films containing GNS@PEG-C, water adsorption was measured also by contact with a filter paper soaked with water at pH 7.2 and at 37 °C, in order to mimic real-use conditions. In these conditions, both **film-GNS-C** and **filmMIX-Ag/GNS-C** absorb water in the quantity of ~17% of their mass in 1 h. Longer contact times up to 24 h lead to minor increases of absorbed water (18–20%).

**Figure 6:**
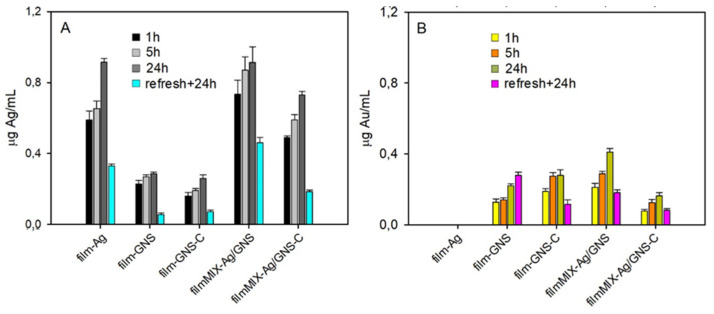
(**A**) Ag release (mg/mL) from 90 mg films fragments in 3.0 mL bidistilled water after 1, 5 and 24 h; ‘refresh +24 h’ data refer to films removed from water after 24 h and dipped in fresh bidistilled water for further 24 h and (**B**) same for Au.

Silver and gold release from films were measured vs. time. Portions of films (90 mg) were dipped in 3 mL bidistilled water inside stoppered vials that were analysed after 1, 5 or 24 h, by carefully taking 1 mL portion of the supernatant solution. Released Ag is reported as grey scale bars in Figure 6A and Au is reported as earth tones scale bars in Figure 6B. The films that remained in the vials for 24 h were carefully removed and dipped in 3 mL of fresh bidistilled water, and the released metals were analysed again after further 24 h (refresh + 24 data, azure and pink bars in Figure 6A,B for Ag and Au, respectively).

Silver is released in small but significant quantities from all films, increasing with time and reaching ~1 ppm concentration after 24 h only in the case of **film-Ag**, **filmMIX-Ag/GNS** and **filmMIX-Ag/GNS-C,** i.e., the three films containing AgNP. The much smaller quantities of silver released from **film-GNS** and **film-GNS-C** are due to the release of the silver metal incorporated in the gold lattice. We expect that the form in which silver is released is Ag^+^, formed by slow AgNP oxidation by O_2_, as it was observed for AgNP immobilized on solid phases [4,54]. In those cases, a steep increase of Ag^+^ was found in the receiving solution in the first 24 h and on longer times, only further minor Ag^+^ release was observed. This fits with the refresh+24h data of Figure 6A, showing that only small quantities of Ag are released from the depleted films after the first 24 h time lap. The quantity of silver released in 1, 5 and 24 h is 15.0%, 17.1% and 23.9% of the total Ag content, respectively, from **film-Ag**, and it is 9.0%, 10.9%, 11.4% and 5.8%, 6.9% and 8.5% from **fimMIX-Ag/GNS** and **filmMIXAg/GNS-C**, respectively. In the case of **film-Ag**, such percent values are slightly higher with respect to what was observed for surface-immobilized AgNP of the same dimensions (that released 5–15% Ag in 24h) [4,54]. In addition, it must be considered that not all AgNP are directly exposed to the water interface, as they are embedded in the film bulk. Moreover, in films containing GNS, despite GNS cannot be oxidized by O_2_, also Au is found in the receiving phase (although in much smaller quantities than Ag, Figure 6B). In particular, the maximum observed Au release is 0.41 ppm after 24 h for **filmMIX-Ag/GNS** (corresponding to 1.2% of total Au). The unexpected Au release could be explained by considering that noble metal cations are adsorbed on the surface of their nanoparticles during synthesis [23,24] and can be released when in contact with pure water. However, a leakage of trace quantities of AgNP and GNS from films could obviously be also hypothesized, especially considering the partial films degradation observed for long dipping times in water. To better investigate this aspect, we measured the Ag and Au release through a dialysis membrane for the representative **filmMIX-Ag/GNS-C**. A 90 mg portion of film was dipped in 5 mL bidistilled water inside a dialysis tube (mwco = 14,000), and the tube was immersed in 12 mL bidistilled water inside a stoppered vial. Further, 24 h were allowed for equilibration. Under these conditions, the free ions concentrations inside and out of the dialysis membrane are identical [55], while the external solution cannot be reached by the large AgNP and GNS. We found 2.5 × 10^−7^ M Ag and 1.8 × 10^−7^ M Au outside the dialysis membrane. This corresponds to the release of 1.8% of total film Ag and of 0.54% of total film Au. Direct release in water from **filmMIX-Ag/GNS-C** (data in Figure 6) gave 8.5% and 0.45% for Ag and Au, respectively. These data suggest that Au cations (most probably Au^+^) are actually released from GNS, while GNS leakage is unlikely. On the other hand, the low measured free Ag^+^ out of the dialysis membrane suggests that AgNP that are smaller than GNS and not directly participating to polymer reticulation may leak from the films. However, it must also be stressed that no LSPR bands of AgNP are seen in the absorption spectra of the receiving solutions in direct release experiments. We can safely state that if AgNP are released from films, this happens only for trace quantities.

### 3.5. Photothermal Effect

**Figure 7:**
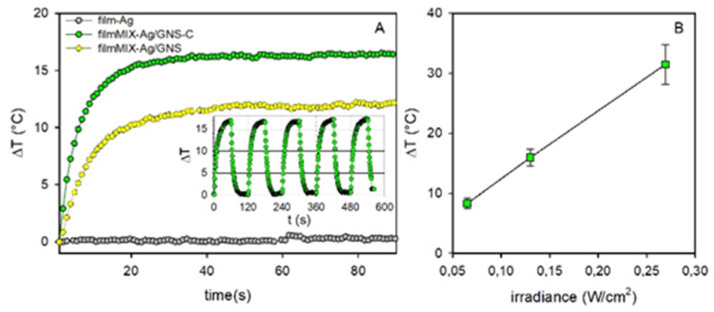
(**A**) Thermograms (ΔT vs. time), irradiance 0.13 W/cm^2^; inset: thermogram for filmMIX-Ag/GNS-C on alternating 60 s irradiation ON with 60 s OFF. (**B**) ΔT vs. irradiance for filmMIX-Ag/GNS-C showing a linear dependence.

Figure 7 displays the photothermal response of **filmMIX-GNS**, **filmMIX-Ag/GNS-C** and **film-Ag**. Irradiation was carried out with a laser source at 800 nm, at irradiances lower than the 0.32 W/cm^2^ limit imposed by ANSI (American National Standard Institute) for the use of laser on human skin at such wavelength [14,15]. The temperature was measured on freshly prepared, dry films using a thermocamera, following an established protocol (see Materials and Methods). At all the used irradiances, we observed a steep T increase in films containing GNS, turning into a plateau after 30–40 s.

The T increase is due to irradiation of the NIR absorption band of the embedded GNS. The different observed ΔT values fit with the different absorption at 800 nm of the two films, see Figure 3B,C. The obtained values are in a range comparable with what already observed on GNS on the surface of glass slides [21] or embedded under a SiO_2_ nanolayer [31] at similar irradiances values. On the contrary, when irradiating **film-AgNP** no T increase was observed (Figure 7A), as AgNP do not have LSPR absorptions over 500 nm (see Supplementary Materials, Figure S5). Figure 7B shows the ΔT value vs. irradiance for **filmMIX-Ag/GNS-C**, displaying the expected, typical linear trend (ΔT measured at the plateau). The inset of Figure 7A displays the thermal response of **filmMIX-Ag/GNS-C** during five cycles of irradiation ON (60 s) and OFF (60 s), showing a perfect stability and reproducibility under the examined conditions. Comparable T increase values (not shown) were measured on **film-GNS** and **film-GNS-C**. As for **film-Ag**, also **film-blank** and **film-PEC** do not have a photothermal response when they were laser-irradiated at 808 nm. While control experiments repeated within 2 days gave identical photothermal responses, control experiments on films aged 30 days showed consistently lower ΔT (~70% decrease) in the case of **filmMIX-Ag/GNS**. In the case of **filmMIX-Ag/GNS-C**, only a ~15% ΔT decrease was observed instead, consistent with the variation of the absorption spectra shown in Figure 3.

### 3.6. Antibacterial and Biocompatibility Studies

The effect of all films was studied on *E. coli* and *S. aureus* planktonic colonies, by dipping 90 mg film portions in 3 mL of cell suspensions in PBS (see Materials and Methods for details). Cell viability was evaluated for immersion times of 5 and 24 h by sampling a small volume of the suspensions and counting the colony forming units (CFU) after incubation for 24 h in Tryptone soya agar. These were compared with the CFU counted on control suspensions, i.e., after the same times but with no added films. The microbicidal effect, ME, is expressed in Equation (1), Results are summarized in Table 2.

Besides the expected finding that ME is larger after 24 h than after 5 h, it must be stressed that the highest ME values are found for both strains at 24 h for two films containing AgNP, i.e., **film-Ag** and **filmMIX-Ag/GNS**. With these two films, we observed ME > 7 at 24 h, meaning that ~0 CFU were found after such exposure time. On the other hand, the significant ME exerted by **film-blank** and **film-pec** is surprising, as both PVA and pectin are known to have no bactericidal properties. To investigate this effect, we checked the ME of all components of **film-blank** and **film-pec,** i.e., PVA, pectin, PEG200 and citric acid, by preparing 3 mL solutions of each of these substances in PBS, at such concentrations as if the 90 mg films samples used for the ME experiments released 100% of their components. The observed ME was null in all cases. We conclude that the ME observed for **film-blank** and **film-PEC** is purely mechanical, i.e., due to the removal of planktonic bacteria from solution, caused by adhesion to the films. To support this conclusion, we contrived a contact-effect experiment for **film-blank**. Microbial suspensions were filtered on cellulose acetate membranes with a porosity of 0.22 μm. The membranes were then deposited on Petri plates containing Tryptone soya Agar (TSA) suitable for the growth of the selected microorganisms and covered with **film-blank**. A membrane without **film-blank** contact was the control. After 5 h contact, the filter membranes were recovered and suspended in sterile water, and the microbial suspensions were then diluted and plated in TSA in order to count the viable cells and calculate the microbicidal effect (24 h contact was also attempted, but partial film disruption was observed, preventing complete detachment from the membrane). Apparent ME values of 1.57 and 1.53 were found for *E. coli* and *S. aureus*, respectively, thus confirming the tendency of both strains to adhere to the PVA films.

To further interpret the ME values of Table 2, it must be remembered that the GNS-containing films release Ag^+^ even when they do not contain AgNP, thanks to Ag contained in the GNS lattice (see Figure 6 for release data). This explains the ME on both strains reported in Table 2 for **film-GNS** and **film-GNS-C**. However, the ME of **film-GNS-C** is much lower than that of **film-GNS**. The same trend is observed also when comparing the ME of **filmMIX-Ag/GNS-C** with that of **filmMIX-Ag/GNS**. The films in which GNS-C acts as reticulant have a distinctly lower ME than their equivalent with GNS and citric acid. As we have verified that citric acid has no antibacterial effects (at least in the range of concentrations possible for these experiments) and as Ag^+^ release is similar in **film-GNS** vs. **film-GNS-C** and in **filmMIX-Ag/GNS** vs **filmMIX-Ag/GNS-C**, we assign the different ME to the more compact texture of the films containing GNS-C and also to the negative charge imparted by the –COO^−^ groups not involved in reticulation: both factors decrease the bacterial adhesion to the film, and, consequently, the additional, apparent ME effect is not observed on planktonic strains. Comparison of SEM images taken on films dipped for 5 h in a suspension of planktonic *E. coli* supports the hypothesis (see Supplementary Materials Figure S8): we found a highly colonized **film-GNS** (Figure S8a) while few, sparse bacteria adhering to **film-GNS-C** (Figure S8b) similarly to *E. coli* adhesion on a plain glass slide, Figure S8c. To complete this analysis, also **film-Ag** (Figure S8d) and **film-blank** (Figure S8e) underwent the same treatment. We found highly colonized films in both cases, similarly to the result with **film-GNS**.

**Figure 8:**
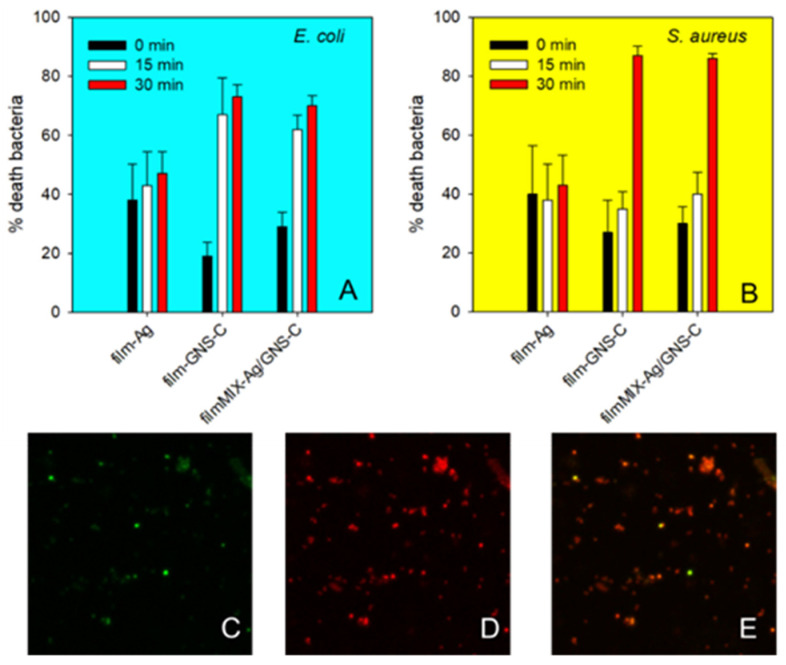
(**A**) Percent fraction of death of *E. coli* bacteria inoculated in the films, with no irradiation (black bars) and laser irradiated at 808 nm for 15 min (white bars) and 30 min (red bars) and (**B**) same, for *S. aureus*. (**C**) Green channel (living cells) in stained **filmMIX-Ag/GNS-C** inoculated with *E. coli* and irradiated for 30 min; (**D**) same, red channel (death cells); and (**E**) same, both channels.

To examine the “on-demand” photothermal action of the films when in contact with bacteria, we followed a method recently proposed by us [39], in which a volume of the planktonic bacterial suspension is inoculated in the film and then is laser treated (details in the Materials and Methods section). After gentle air drying, the inoculated spot was irradiated for different times (15 and 30 min) with an 800 nm laser source at a 0.30 W/cm^2^ irradiance. Inoculated, non-irradiated samples were also examined, as control. The samples were then stained with the Film Tracer Live/Dead viability kit (L10316, Invitrogen), which stains viable bacteria in green and dead ones in red (i.e., with damaged membranes). Confocal laser scanning microscope (CLSM) analysis allowed to determine the percent area in the green and red channels in the treated area and, consequently, the percent of living and dead bacteria. **Film-Ag**, **film-GNS-C** and **filmMIX-Ag/GNS-C** were selected for analysis, together with **film-blank** (reference). Results are summarized in Figure 8A,B; panels (Figure 8C–E) are representative CLSM images of the green, red and mixed channels, respectively, in the case of *E. coli* inoculated on **filmMIX-Ag/GNS-C** and irradiated for 30 min. Noticeably, also in experiments carried out on **film-blank** (not included in Figure 8), a fraction of dead bacteria in the 21–26% death range was found for both strains and with no correlation with 0, 15 or 30 min irradiation time. This is to be attributed to the inherent stress conditions of the experiment set up, as, when inoculated in the films, bacteria are at room temperature, in a nutrient-free environment. Further control experiments with an inert surface (planktonic bacteria dropped on glass) gave similar dead bacteria fractions.

However, Figure 8 clearly shows that, with both strains, for films containing GNS (thus displaying a photothermal effect), an increased death fraction is observed on laser irradiation. The percent death fraction also increases on increasing irradiation time. In particular, ~80% death fraction is reached with both strains and both **film-GNS-C** and **filmMIX-Ag/GNS-C** after 30 min irradiation. In the case of *E. coli*, a lower but comparable death fraction is found also after 15 min irradiation. With *S. aureus*, 30 min irradiation is needed to observe an effect different with respect to non-irradiated film. This is coherent with the higher resistance of Gram-positive bacterial strains with respect to Gram-negative ones, due to the peptidoglycan rigid cellular walls of the former [56]. With **film-Ag**, irradiation does not give a T increase. As expected, no significant increase of death fraction is observed on irradiation. However, the intrinsic bactericidal effect of film-AgNP can be seen in the death fractions for both *E. coli* and *S. aureus*. These are higher than what found for the GNS-containing films when non-irradiated, even in the case of **filmMIX-Ag/GNS-C**. We believe that this is related to the tendency of **film-Ag** to release larger Ag^+^ quantities with respect to the AgNP embedded in **filmMIX-Ag/GNS-C**, due to the less compact structure of the former. This is also evidenced in Figure 6, if shorter Ag^+^ release time (1 h) is compared for the two films. As a whole, the data collected in the irradiation experiments show that the photothermal effect adds a further killing action along the intrinsic one, on both the examined colonies, at least if 30 min irradiation time is used.

Finally, we have also verified the biocompatibility of all films on human fibroblasts, using an indirect test. 1 cm^2^ of the chosen film was soaked with 2 mL of complete culture medium (CM), at 37 °C, for 24 h. Then, 200 μL of this conditioned culture medium were put in contact with cells, again for 24 h, at 37 °C (details in the Materials and Methods section). CM was used as the reference (100% viability). Results (Supplementary Materials Figure S9) show a ~100% viability for **film-blank**, **film-PEC**, **film-GNS** and **film-GNS-C**. Only two AgNP-containing film, **film-Ag** and **filmMIX-Ag/GNS**, cause a modest adverse effect, with fibroblasts viability reduction to 88% and 92%, respectively. In the case of **filmMIX-Ag/GNS-C,** 97% viability was found, most probably in connection to the lower total silver release found after 24 h (Figure 6A). These viability data allow to assess a reassuring biocompatibility of all the examined films.

## 4. Conclusions

We found the correct synthetic conditions to allow AgNP and GNS to coexist in the same precursor solution, from which PVA films containing both nanoparticles were obtained. A set of films with separate or mixed AgNP and GNS was prepared, using either citric acid or the terminal -COOH groups of HS-PEG-COOH as reticulant when this was the GNS coater. Noticeably, films containing both AgNP and GNS are a novelty in the literature. All films containing AgNP, GNS or both revealed of being capable of exerting an intrinsic, prolonged antibacterial action (increasing from 5 to 24 h), due to the release of Ag^+^. The latter can come from the lattice of GNS (in which Ag is included in low percent during their synthesis) and from AgNP. However, in films with embedded AgNP, the 5 and 24 h microbicidal action was noticeably stronger. Adhesion and bacteria subtraction from planktonic suspensions were also noticed, particularly for the less compact films reticulated with citric acid, leading to an additional but only apparent microbicidal effect. Furthermore, we demonstrated that along the intrinsic effect, an on-demand photothermal antibacterial action is obtained with laser irradiation, for the films containing GNS. As we have observed that the GNS NIR absorption band of **filmMIX-Ag/GNS** (citric acid as reticulant) significantly decays in a 30 days lapse, while it remains intact in **filmMIX-Ag/GNS-C**, we conclude that the latter could be an excellent candidate as a bifunctional medical device, for practical applications such as wounds disinfection or healing. With this film, an authentic > 4 log units CFU reduction (ME) is observed in 24 h both for planktonic *E. coli* and *S. aureus*, thanks to the intrinsic action of silver, while the large majority of the bacteria in direct contact with the film can be killed if laser treatment as short as 30 min is applied. Such treatment takes place at 800 nm, i.e., with a low energy radiation inside the biotransparent window, and at irradiances values (0.3 W/cm^2^) well under the limit imposed by ANSI for human skin. Finally, the suitability of this film for real use on wounds is also supported by its excellent biocompatibility, as observed in cytotoxicity tests on human fibroblasts.

## Figures and Tables

**Table 1:** Composition of the film-forming solutions.

	PECTIN ^1^	AgNP ^2^	GNS@PEG ^3^	GNS@PEG-C ^4^	CA ^5^	TOTAL Ag ^6^(MOL; μg)	TOTAL Au ^7^(MOL; μg)	CALCULATED FILM MASS ^8^ (mg)	DRY FILM MASS ^9^ (mg)
FILM-BLANK	-	-	-	-	✓	-	-	732	731(±5)
FILM-PEC	1.0	-	-	-	✓	-	-	742	740(±8)
FILM-Ag	-	1.0	-	-	✓	8.82 × 10^−7^ 95.1	-	742	748(±10)
FILM-GNS	-	-	0.5	-	✓	9.7 × 10^-7^ 104	4.34 × 10^−6^ 855	733	728(±12)
FILM-GNS-C	-	-	-	0.5	-	9.3 × 10^-7^ 99	4.26 × 10^−6^ 840	667	660(±11)
FILM-MIX-Ag/GNS	-	1.0	0.5	-	✓	1.85 × 10^−6^ 199	4.34 × 10^−6^ 855	743	746(±9)
FILM-MIX-Ag/GNS-C	-	1.0	-	0.5	-	1.81 × 10^−6^ 194	4.26 × 10^−6^ 840	677	686(±8)

^1^ mL of 1% **w*/*w** pectin solution added to 10 mL of PVA solution; ^2^ mL of AgNP colloidal solution added to 10 mL PVA solution (please note that AgNP are synthesized in 1% *w*/*w* pectin solution); ^3^ mL of concentrated GNS@PEG solution added to 10 mL PVA solution; ^4^ same, for GNS@PEG-C; ^5^ the **✓** symbol indicates in which cases 66 mg of citric acid were added to the PVA solution; ^6^ total Ag in the film-forming solution (considering both added AgNP and the Ag quantity contained in GNS); ^7^ total Au quantity in the film-forming solution; ^8^ sum of the mass of all added solid components; ^9^ actual weight after preparation and 4 days drying at RT.

**Table 2:** ME at 5 and 24 h on planktonic *E. coli* and *S. aureus* ^1^.

	***E. coli***	
	**5 h**	**24 h**
**Film-blank**	1.2(0.2)	2.3(0.2)
**Film-pec**	1.7(0.3)	3.0(0.2)
**Film-Ag**	5.5(0.3)	> 7
**Film-GNS**	2.2(0.2)	4.9(0.3)
**Film-GNS-C**	0.8(0.1)	2.3(0.2)
**FilmMIX-Ag/GNS**	5.6(0.3)	>7
**FilmMIX-Ag/GNS-C**	0.2(0.1)	4.4(0.3)
	***S. aureus***	
	**5 h**	**24 h**
**Film-blank**	2.9(0.2)	3.2(0.2)
**Film-pec**	3.1(0.2)	3.7(0.3)
**Film-Ag**	3.1(0.3)	>7
**Film-GNS**	3.7(0.2)	4.6(0.3)
**Film-GNS-C**	1.1(0.2)	2.3(0.2)
**FilmMIX-Ag/GNS**	3.5(0.3)	>7
**FilmMIX-Ag/GNS-C**	1.3(0.2)	4.7(0.3)

^1^ Reported data are the average of 3 experiments; standard deviation is given in parentheses.

## Supplementary Materials

The following are available online at https://www.mdpi.com/article/10.3390/nano11061387/s1, Figure S1: Additional TEM images, Figure S2–S6: Additional absorption spectra of solutions and films (including evolution with time), Figure S7: Additional SEM images, Figure S8: SEM images of adhering *E. coli*, Figure S8: Fibroblasts vitality results.
