# Supplementary material for: PVA Films with Mixed Silver Nanoparticles and Gold Nanostars for Intrinsic and Photothermal Antibacterial Action
Source: Nanomaterials (Basel). 2021 May 25;11(6):1387. doi: 10.3390/nano11061387 (PMC8225135; doi:10.3390/nano11061387)
Supplement: Supplementary file 1 [file nanomaterials-11-01387-s001.zip › nanomaterials-1214345-supplementary.pdf]

SUPPLEMENTARY MATERIALS

PVA films with mixed silver nanoparticles and gold nanostars for intrinsic and photothermal antibacterial action

S1 – Additional TEM images

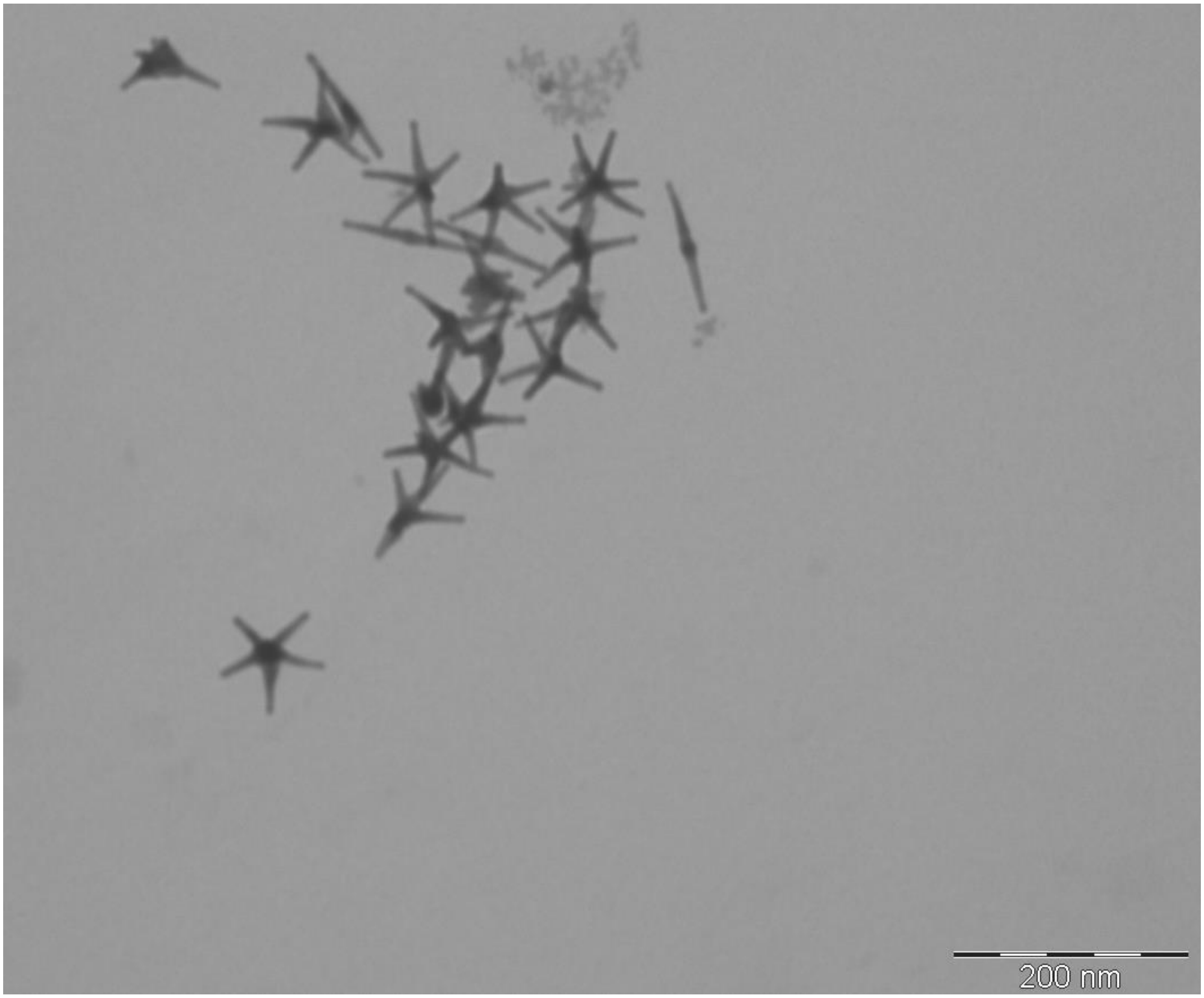

Figure S1a – GNS@PEG

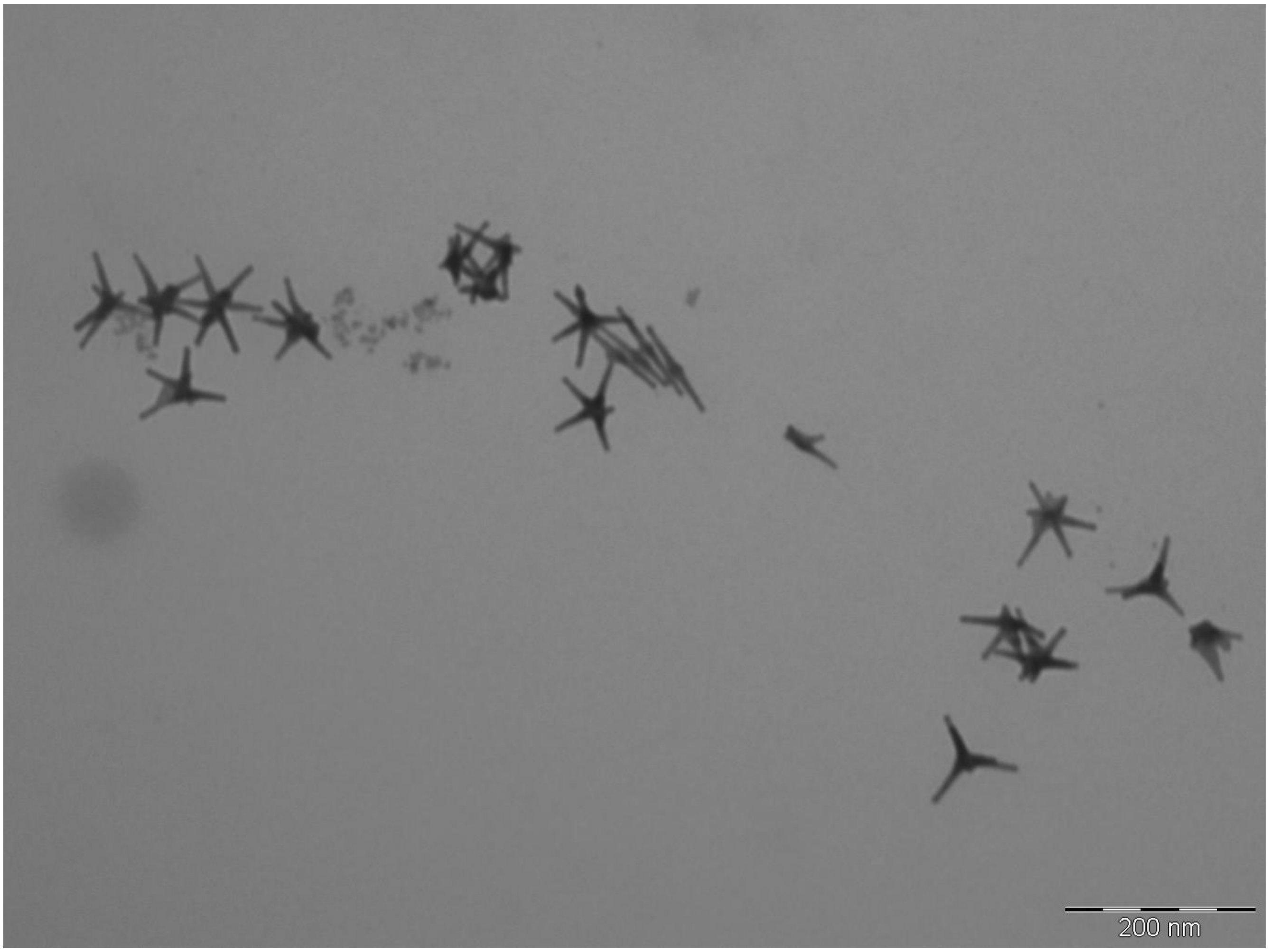

Figure S1b – additional TEM image of GNS@PEG

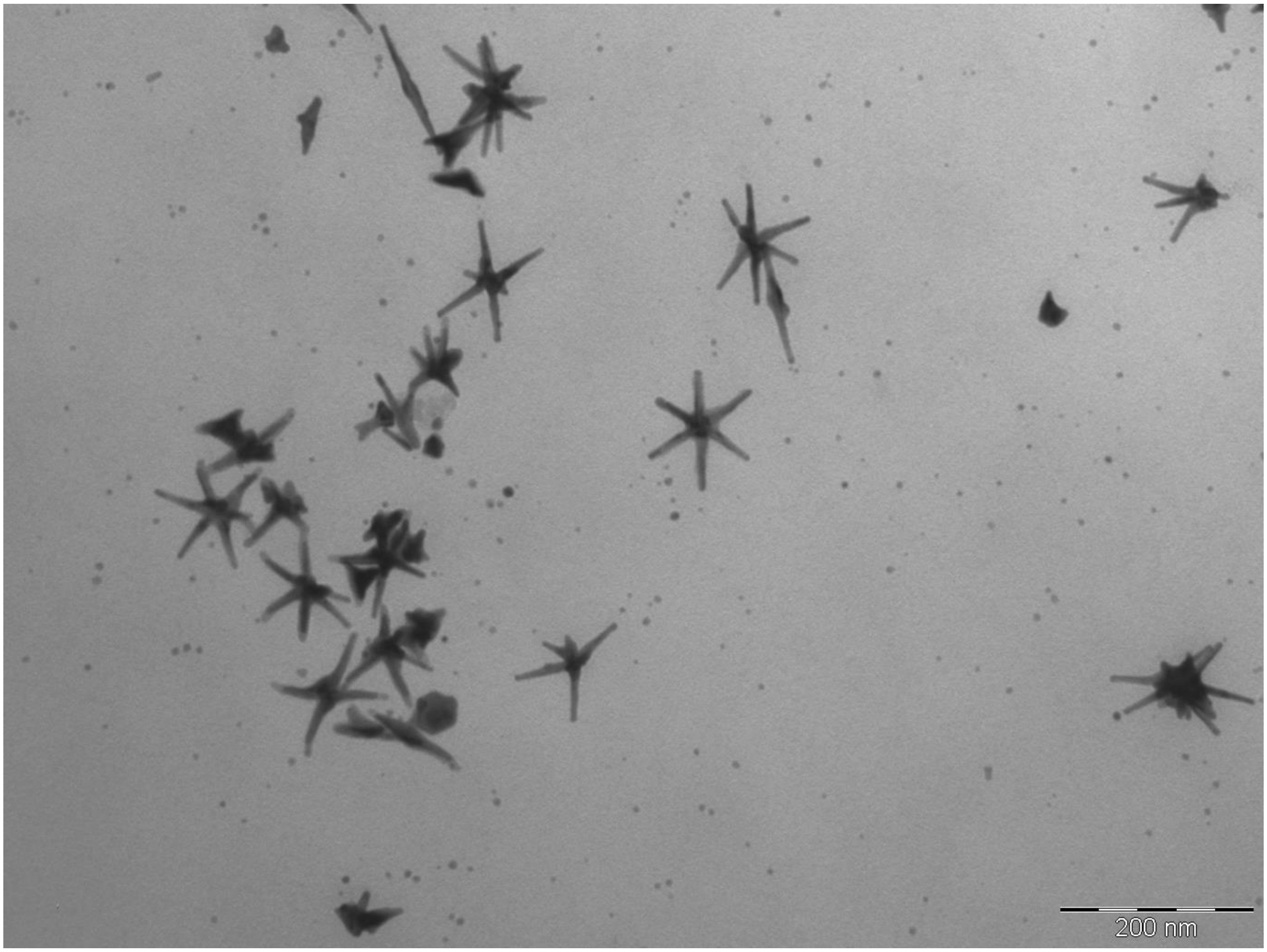

Figure S1c – TEM image from mixed AgNP/GNS@PEG solution

S2 – Absorption spectra of an AgNP solution with GNS additions

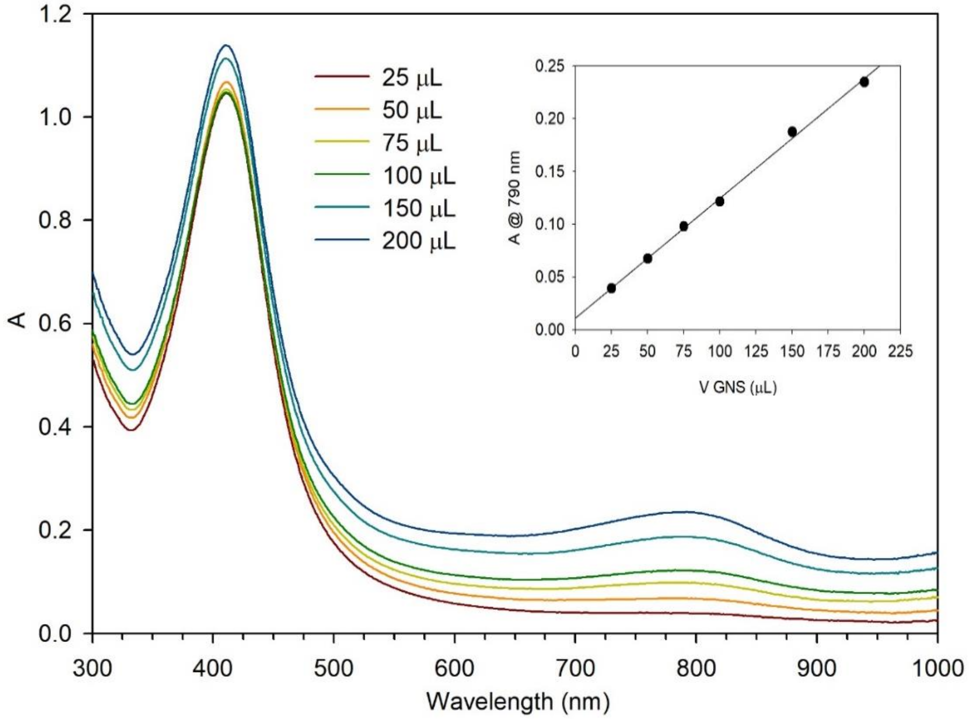

Figure S2: Absorption spectra of an AgNP solution (3.0 mL) to which increasing quantities of a concentrated GNS@PEG solution were added. A linear Abs vs GNS added volume is observed (inset) at 790 nm (absorption maximum of the GNS solution used in this experiment).

S3 – GNS (@PEG) and mixed AgNP/GNS@PEG solutions

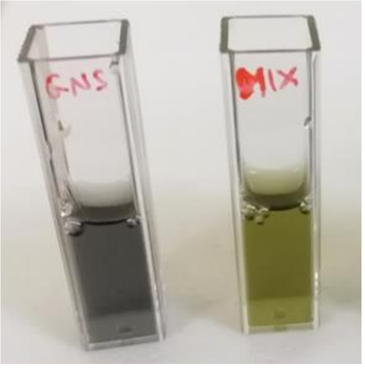

Figure S3a: photographs of a GNS@PEG solution (left) and of a mixed AgNP/GNS@PEG (2:1 v/v) solution (right)

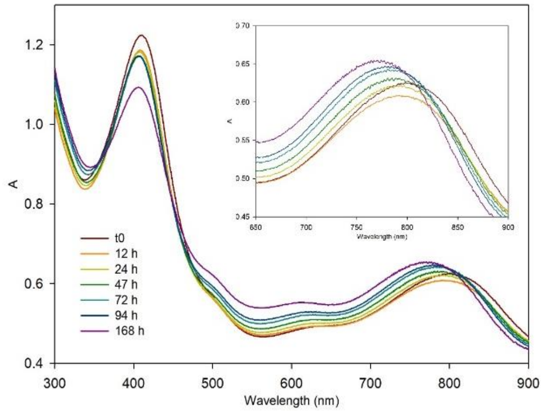

Figure S3b: absorption spectra of an as prepared AgNP (pH 10.9) mixed with a GNS@PEG solution (3:1 v/v) vs time: in 7 days the LSPR absorption of AgNP (410 nm) decreases, and the LSPR absorption of GNS (800 nm ca) shifts to shorter wavelengths (see also inset)

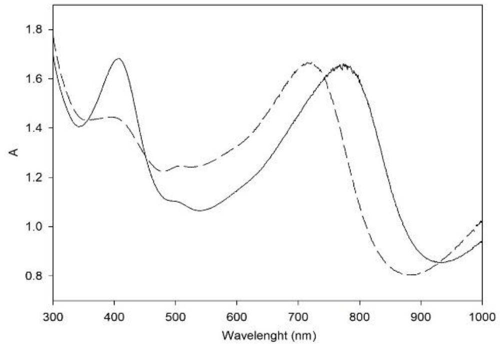

Figure S3c: absorption spectra of an as prepared AgNP (pH 10.9) mixed with a GNS@PEG solution (2:1 v/v) just mixed (solid line) and after 35 days (dashed line)

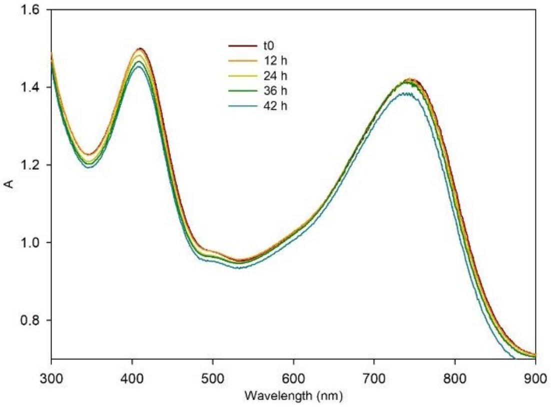

Figure S3d: absorption spectra with time of an AgNP solution after neutralization (pH 7.1) mixed with a GNS@PEG solution (2:1)

S4 – mixed solution of citrate coated AgNP and non-pegilated GNS

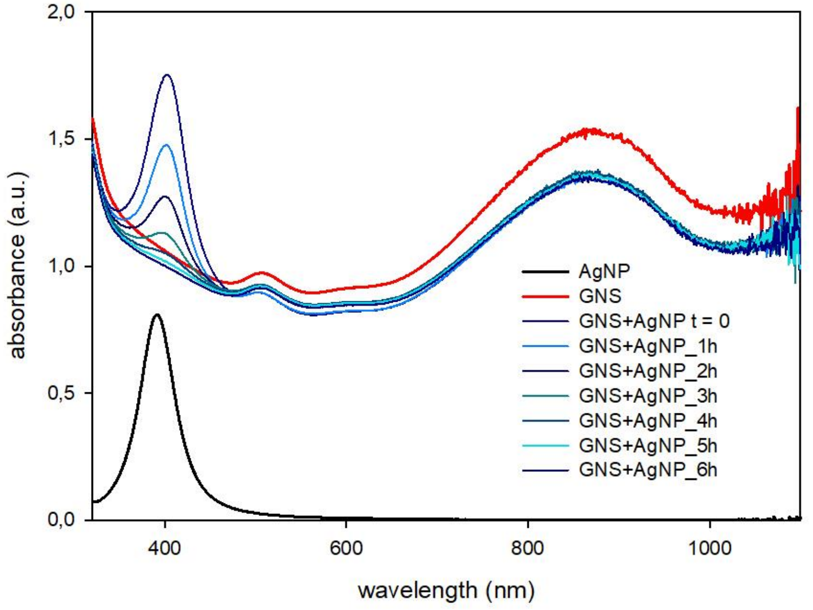

Figure S4: in 7 days the AgNP LSPR band (ca 400 nm) completely disappears, while the GNS band (ca 850 nm) shifts to the blue. The absorption spectrum of the citrate coated-AgNP solution is also reported for comparison (lower black line)

S5 – Absorption spectra of AgNP in the presence of all film components except GNS

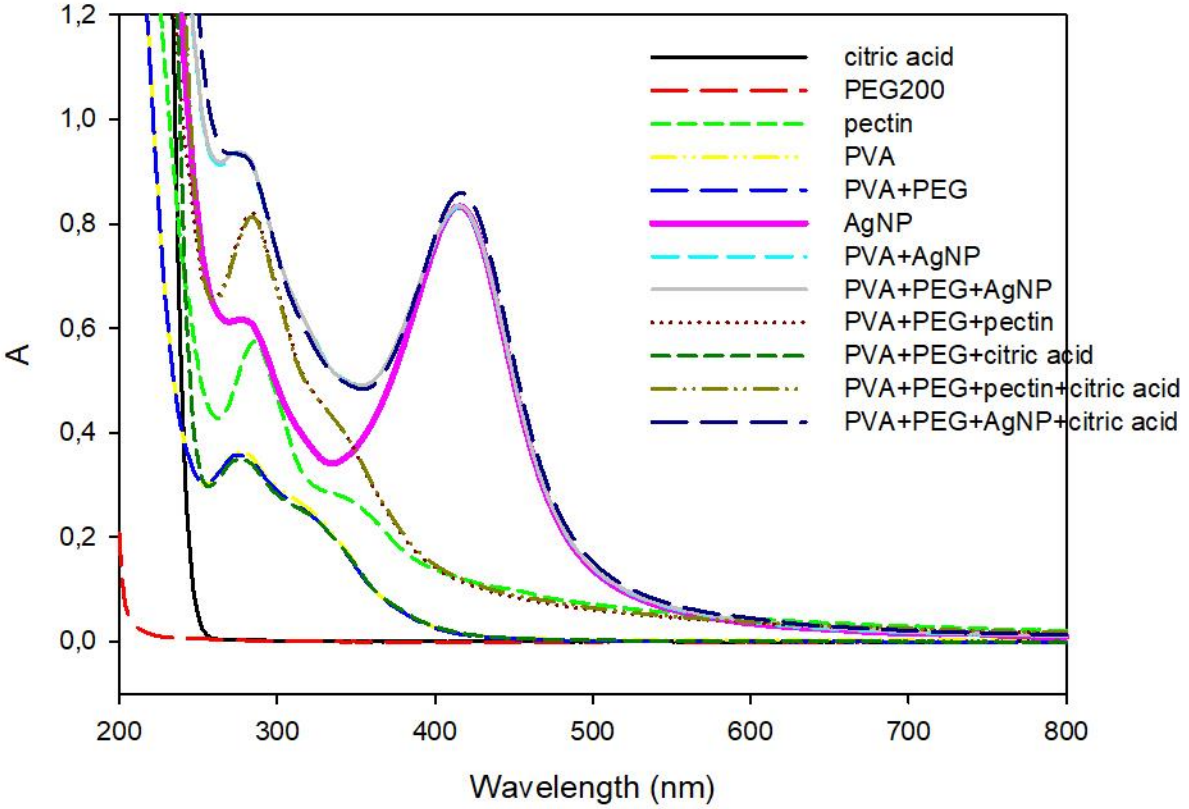

Figure S5 – absorption spectrum of an AgNP solution alone (thick pink spectrum) and after addition of PVA and of all film components except GNS. The absorption spectra of PVA, PEG200 pectn and citric acid have also been added to the figure, for comparison

S6 – Absorption spectra of film-GNS and film-GNS-C, 30 days aged

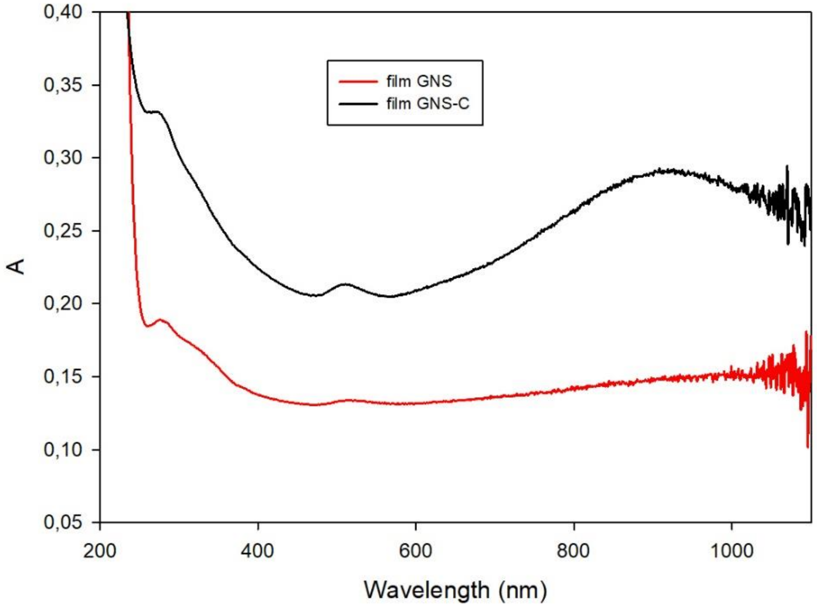

Figure S6: absorption spectra after 30 days ageing. Black: film-GNS-C. Red: film-GNS (in the latter case, the GNS LSPR band is flattened and strongly decreased in intensity)

S7 – SEM image of filmMIX-Ag/GNS-C

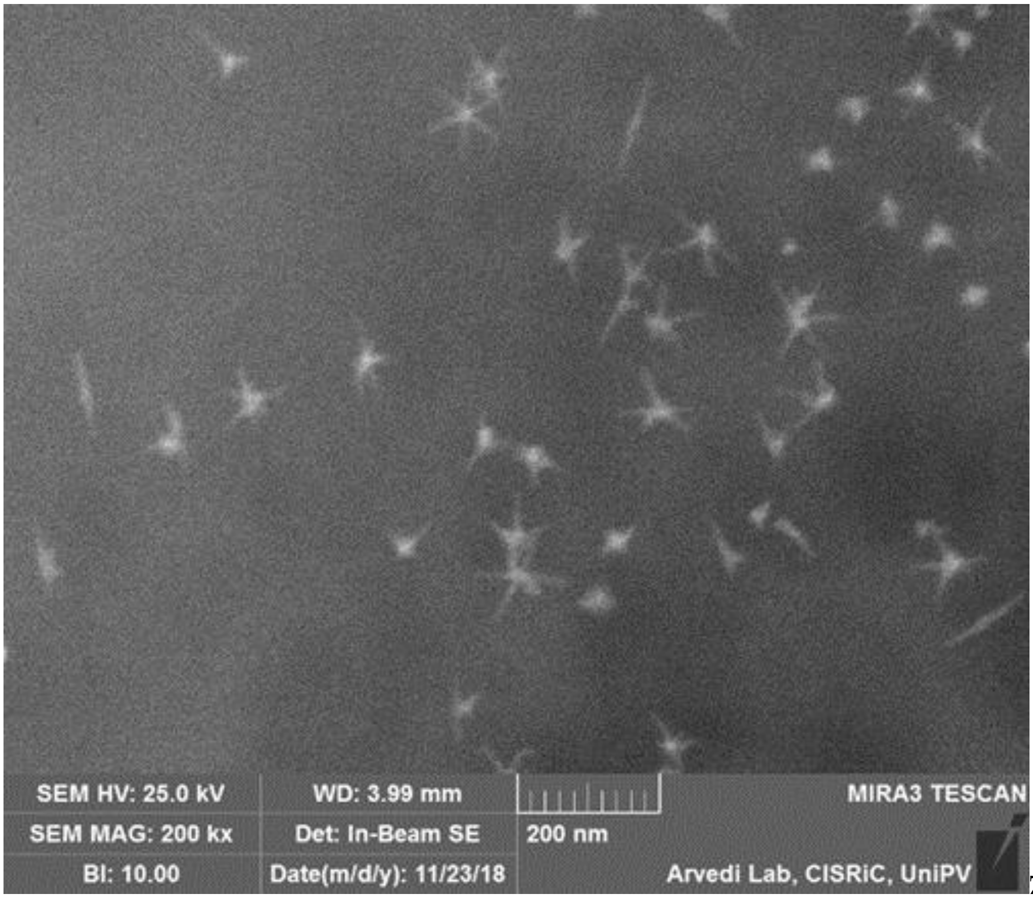

7

Figure S7. SEM image (200.000 x) of filmMIX-Ag/GNS-C

S8 – SEM images of E. coli bacteria adhering to surfaces

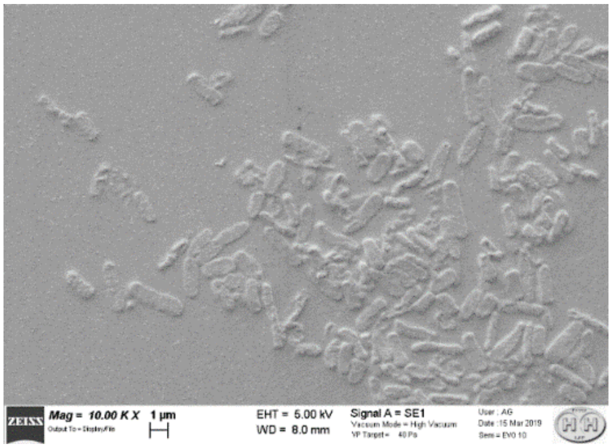

Figure S8a – Adhesion of *E. coli* bacteria on **film-GNS**

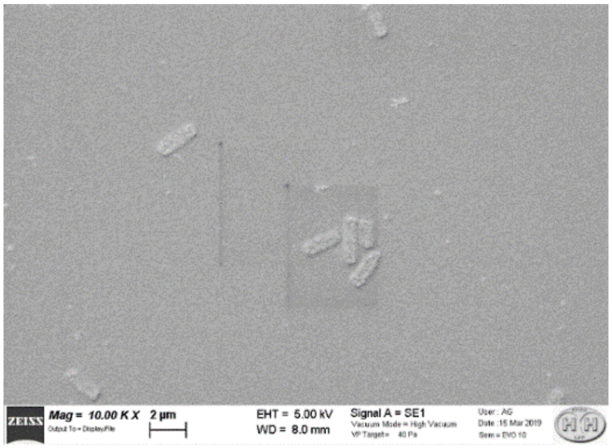

Figure S8b – Adhesion of *E. coli* bacteria on **film-GNS-C**

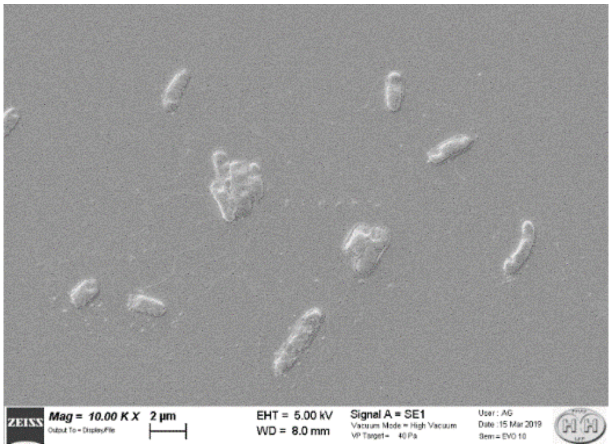

Figure S8c – Adhesion of *E. coli* on a plain glass slide

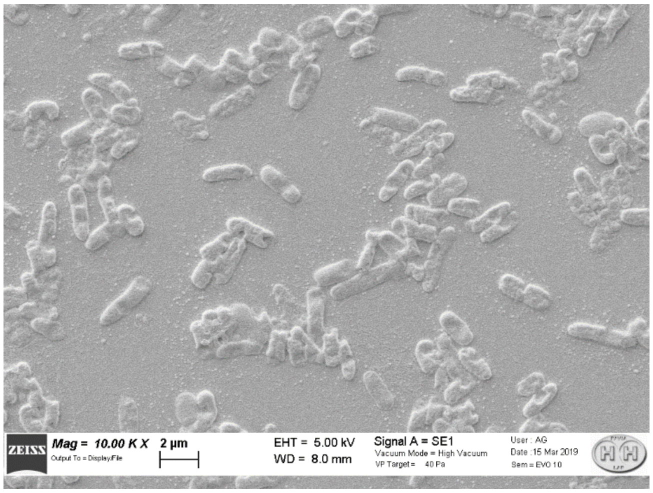

Figure S8d – Adhesion of *E. coli* on **film-Ag**

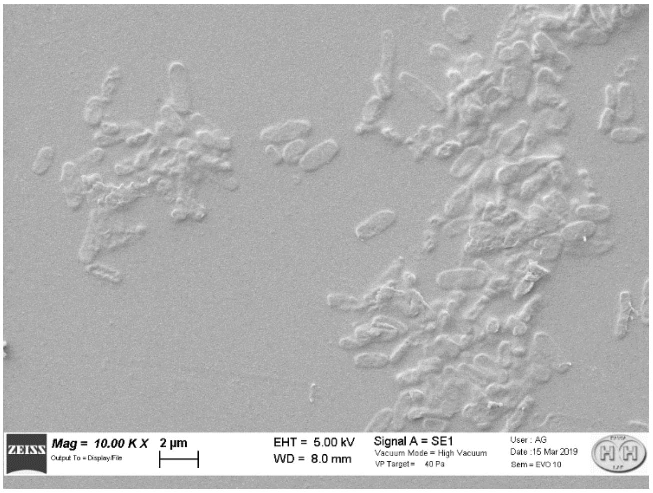

Figure S8e – Ashesion of *E. coli* on **film-blank**

S9 – Interaction with fibroblasts

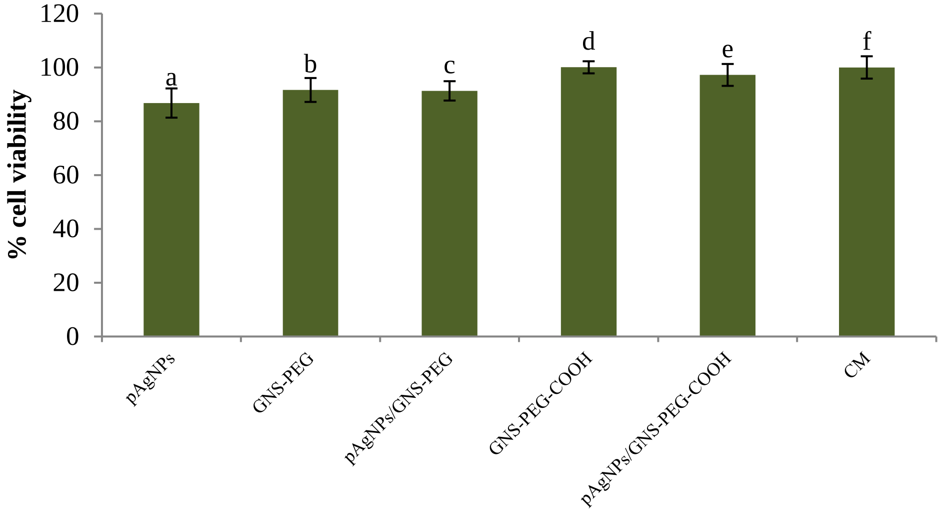

Figure S9: Values of cell (Human Fibroblasts) viability %; CM was considered as reference (mean values ± s.d.; n=12). Anova one-way – Multiple Range Test ($p<0.05$): a vs d-f; b vs d-f; c vs d-f.
